# Causal Associations between Gut Microbiota and Different Types of Dyslipidemia: A Two-Sample Mendelian Randomization Study

**DOI:** 10.3390/nu15204445

**Published:** 2023-10-20

**Authors:** Xuyi Zhou, Peiqi Lian, Hui Liu, Yinghui Wang, Meijuan Zhou, Zhijun Feng

**Affiliations:** Department of Radiation Medicine, Guangdong Provincial Key Laboratory of Tropical Disease Research, NMPA Key Laboratory for Safety Evaluation of Cosmetics, School of Public Health, Southern Medical University, Guangzhou 510515, China; zhouxuyi1323@163.com (X.Z.); kyson0329@foxmail.com (P.L.); lhuidoc@163.com (H.L.); yinghui0618@163.com (Y.W.)

**Keywords:** dyslipidemia, gut microbiota, causal associations, mendelian randomization study

## Abstract

The determination of a causal association between gut microbiota and a range of dyslipidemia remains uncertain. To clarify these associations, we employed a two-sample Mendelian randomization (MR) analysis utilizing the inverse-variance weighted (IVW) method. This comprehensive analysis investigated the genetic variants that exhibited a significant association (*p* < 5 × 10^−8^) with 129 distinct gut microbiota genera and their potential link to different types of dyslipidemia. The results indicated a potential causal association between 22 gut microbiota genera and dyslipidemia in humans. Furthermore, these findings suggested that the impact of gut microbiota on dyslipidemia regulation is dependent on the specific phylum, family, and genus. Bacillota phylum demonstrated the greatest diversity, with 15 distinct genera distributed among eight families. Notably, gut microbiota-derived from the Lachnospiraceae and Lactobacillaceae families exhibit statistically significant associations with lipid levels that contribute to overall health (*p* < 0.05). The sensitivity analysis indicated that our findings possess robustness (*p* > 0.05). The findings of our investigation provide compelling evidence that substantiates a causal association between the gut microbiota and dyslipidemia in the human body. It is noteworthy to highlight the significant influence of the Bacillota phylum as a crucial regulator of lipid levels, and the families Lachnospiraceae and Lactobacillaceae should be recognized as probiotics that significantly contribute to this metabolic process.

## 1. Introduction

The gut microecosystem, consisting of approximately 1014 microorganisms [1], is the most extensive, intricate, and vulnerable microecosystem within the human body [2]. It assumes a crucial role in both human health and diseases. Among the various microorganisms present in this microecosystem, the gut microbiota, including bacteria, viruses, fungi, and other microorganisms, is a substantial constituent [3], with bacteria accounting for more than 95% of the overall population [4]. The significance of gut microbiota has been increasingly validated via extensive research. Firstly, the establishment of normal intestinal flora via enteral colonization is imperative for the maintenance of intestinal barrier function [5]. Secondly, gut microbiota bestows various advantages on the host, including intestinal, immune, and nutritional benefits [6], thereby facilitating digestion, regulating gut hormone secretion and physiological development, and defending against pathogen colonization [7,8,9]. The prevailing belief in current research suggests that alterations in the gut microbiota exert a substantial influence not only on the host’s gastrointestinal disorders but also on a diverse array of extraintestinal diseases [10], including diabetes [11,12], obesity [13,14,15,16], chronic kidney disease (CKD) [17,18,19], hyperlipidemia [20], cardiovascular disease [21,22], metabolic disturbances [23], colon cancer [24,25], and other intestinal diseases [26,27]. Furthermore, Furthermore, several scholarly investigations have also assessed the influence of gut microbiota on the regulation of brain behavior and the immune system, encompassing the intestinal nervous system [28], neuroimaging [29], inflammatory diseases [30,31,32,33], and the gut microbiota-intestinal-brain axis [34,35,36,37]. Moreover, individuals can employ flora transplantation to rectify disruptions in the host’s gut microbiota, thereby reinstating its normal and stable state and preserving the host’s intestinal equilibrium [38]. In summary, the intercommunication signals between the host and gut microbiota, encompassing the modulation of host metabolism using the gut microbiota, have the potential to impact the physiological well-being and pathological conditions of the host [39]. Previous scholarly works have extensively explored the potential regulatory importance of the gut microbiota in lipid metabolism disorders [40], thus suggesting that manipulating the gut microbiota may offer a crucial strategy for managing hyperlipidemias [41]. Furthermore, several studies have documented that the regulation of gut microbiota disorder, coupled with the inhibition of abnormal lipid metabolism, holds promise for ameliorating the advancement of liver injury [42]. These findings lend support to the potential impact of gut microbiota on lipid metabolism. Nevertheless, the causal association between gut microbiota and host lipid metabolism disorders remains inconclusive.

Dyslipidemia is presently characterized in clinical settings by the presence of anomalies in various lipid types, including high-density lipoprotein cholesterol (HDL-C), low-density lipoprotein cholesterol (LDL-C), triglyceride (TG), total cholesterol (TC), apolipoprotein A1 (APOA1), and apolipoprotein B (APOB) concentrations [43,44,45,46]. Dyslipidemia can be regarded as a manifestation of lipid metabolism disorders or as a concomitant symptom of multiple diseases, including obesity [47], type 2 diabetes (T2D) [48], CKD [49,50,51], atherosclerosis and coronary heart disease (CHD) [52,53,54], and malignant tumors [55,56,57,58]. It is widely recognized that elevated TG levels serve as not only a risk factor for acute pancreatitis [46] but also an independent “risk-enhancing factor” for atherosclerotic cardiovascular disease (ASCVD) [43,59]. In the context of individuals diagnosed with high or extremely high-risk ASCVD, current guidelines emphasize the necessity of reducing LDL-C levels to the utmost extent in order to mitigate the occurrence of severe complications [60]. The levels of APOB protein have been found to have a positive correlation with hypercholesterolemia, and a decrease in APOB synthesis has been shown to significantly reduce LDL-C levels and the prevalence of atherosclerosis [61,62]. Conversely, high levels of HDL-C have not been firmly established as a risk factor for CHD [63]. APOA1, a crucial component of HDL-C, contributes to over 70% of lipoproteins [64,65,66,67,68], which are also part of the HDL-C family and share a similar physiological function. The aforementioned evidence serves to illustrate the direct influence of lipid levels on the cardiovascular system. Due to the prevalence and significant impact of dyslipidemia on overall health, this study aims to investigate the potential causal associations between gut microbiota and lipid metabolism regulation in order to identify evidence supporting the use of gut microbiota modulation as a strategy for managing lipid metabolic disorders.

Mendelian randomization (MR) analysis is a prevalent approach employed in population studies to evaluate causality, wherein genetic variation is utilized to ascertain the coherence between observed associations linking risk factors and outcomes [69,70]. The selection of genetic variation as an instrumental variable (IV) was employed in the implementation of Mendelian randomization (MR) to establish causality due to the random allocation and lifelong exposure of genetic alleles, thereby mitigating potential confounding factors inherent in the genetic process [71]. Furthermore, the majority of genetic variants frequently lack association with conventional epidemiological risk factors, rendering traditional epidemiological analysis techniques insufficient in accurately elucidating a causal association between genetic variants and diseases [72]. Mendelian randomization offers valuable guidance for investigations reliant on genetic variation, thereby mitigating or circumventing the bias induced by confounding factors inherent in traditional epidemiological methods [73,74,75]. In this present study, an MR analysis was performed on a substantial community sample of European participants to investigate the causal association between various genus-based gut microbiota and dyslipidemia. By employing human genetic data within the MR framework, this study elucidates the impact of distinct gut microbiota genera on different types of dyslipidemia, thereby offering innovative perspectives on the potential causal associations between gut microbiota and dyslipidemia.

## 2. Material and Methods

### 2.1. Exposure Data

Genetic variants that exhibit a robust association with distinct genera of gut microbiota were identified using a comprehensive genome-wide association study (GWAS) conducted on individuals of European descent, as documented in the OpenGWAS database [76,77]. The study’s methodology is visually depicted in Figure S1. We conducted an IV screening using the “TwoSampleMR” R package [74,78,79] to obtain independent IVs that affect lipoprotein levels in various gut microbiota data sets. The parameters used were as follows: p1 = 5 × 10^−8^ (genetic variants must exhibit a strong association with the exposure), clump = TRUE, r^2^ = 0.01, kb = 5000 (IVs with linkage disequilibrium were removed to ensure the independence of the selected genetic variations) [80,81]. A comprehensive screening process was conducted on 129 potential datasets to identify IVs for exposure, with their corresponding GWAS IDs ranging from “EBI-A-GCST90016959” to “EBI-A-GCST90017087” (Table S1).

### 2.2. Outcome Data

SNPs associated with dyslipidemia (HDL-C, LDL-C, TG, TC, APOA1, APOB) were also obtained from the OpenGWAS database, and the population structure is also dominated by European (Table S1). If there were two studies with overlapping data, the study with the largest sample size was included. In this step, we intersected the independent IVs from exposure factors and single nucleotide polymorphisms (SNPs) of outcome event and constructed an association of “independent exposure IV”—“factors”—“outcome variables” and eliminated SNPs associated with potential confounding variables via the PhenoScanner [82,83] database (http://www.phenos-canner.medschl.cam.ac.uk/phenoscanner, accessed on 5 September 2023). If a specific SNP exhibits a direct correlation with lipid abnormalities in the host or with abnormalities in liver function (that may impact lipid synthesis), host fat distribution, fatty acid metabolism, and body mass index (BMI), it is deemed to possess potential confounding effects, leading to its exclusion from the study. Then, we combined the two sets of data for subsequent MR analysis.

### 2.3. Ethics Statement

The present study utilized publicly accessible GWAS summary statistics data sourced from the OpenGWAS database. This database obtained informed consent from all participating studies in accordance with the protocols approved by their respective institutional review boards. Consequently, the submission of a dedicated ethics statement is unnecessary.

### 2.4. Statistical Analysis

The standard inverse-variance weighted (IVW) method was employed for primary two-sample Mendelian randomization (MR) analyses, which were further enhanced by incorporating the weighted median and MR Egger methods available in the TwoSampleMR package [78,84]. The study aimed to examine the variability in the association between different genera of gut microbiota and different types of dislipidemia by utilizing Cochran’s Q statistics [48,85]. Heterogeneity was ascertained by assessing the significance of the *p* value (less than 0.05) derived from the Q statistic. In cases where heterogeneity was present, the effect evaluation was estimated using the random-effects IVW method, while the fixed-effects model was employed in the absence of heterogeneity [86,87]. Sensitivity analyses were conducted to identify and address potential pleiotropy in the causal estimates [88,89,90]. Specifically, we assessed the presence of horizontal pleiotropy using MR-Egger regression, considering its intercept terms and the Mendelian randomization pleiotropy residual sum [78,91]. When the intercept of the MR-Egger model deviates significantly from zero, or its *p* value is less than 0.05, it suggests the presence of horizontal pleiotropy. In such cases, an alternative MR method was employed to report the findings [69,92]. For determining the final results, causal associations were considered statistically significant if the *p* value was less than 0.05.

## 3. Results

### 3.1. Dyslipidemia MR Estimates 

**Figure 1:**
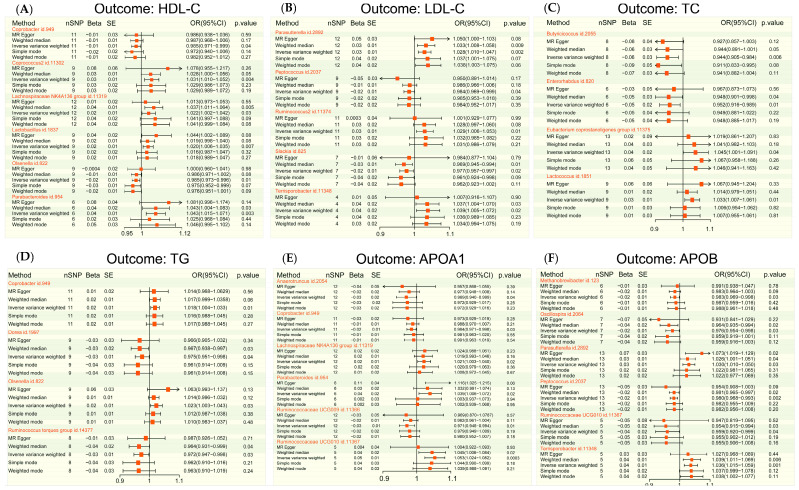
The forest map illustrates the results of Mendelian randomization (MR) analysis, indicating the impact of various gut microbiota genera on different lipid levels. A-F, the MR analysis demonstrates diverse effects of gut microbiota genera on HDL-C (**A**), LDL-C (**B**), TC (**C**), TG (**D**), APOA1 (**E**), and APOB (**F**). nSNP, number of single nucleotide polymorphism; SE, standard error; OR, odds ratio; high-density lipoprotein cholesterol, HDL-C; low-density lipoprotein cholesterol, LDL-C; triglyceride, TG; total cholesterol, TC; apolipoprotein A1, APOA1; apolipoprotein B, APOB.

In the context of two-sample MR Analysis, we have effectively discerned six gut microbiota genera that exhibit causality towards HDL-C (Figure 1A), five towards LDL-C (Figure 1B), four towards TC (Figure 1C), four towards TG (Figure 1D), six towards APOA1 (Figure 1E), and six towards APOB (Figure 1F). It is worth noting that the number of independent IVs employed varied across the different sets of causal associations under investigation. Based on the final results, it is evident that the distribution of gut microbiota genera exhibiting a negative causal association with various forms of dislipidemia (OR < 1, *p* value of IVW < 0.05) can be outlined as follows: Coprobacter and Olsenella for HDL-C (Figure 1A), Peptococcus and Slackia for LDL-C (Figure 1B), Butyricicoccus and Enterorhabdus for TC (Figure 1C), Dorea and Ruminococcus torques group for TG (Figure 1D), Anaerotruncus, Coprobacter, and Ruminococcaceae UCG009 for APOA1 (Figure 1E), and Methanobrevibacter, Oscillospira, Peptococcus, and Ruminococcaceae UCG010 for APOB (Figure 1F). This observation suggests that an increase in the abundance of these bacterial genera in the gut is associated with a decrease in the production of the corresponding lipids. In contrast, the distribution of gut microbiota genera that exhibit a positive causal association with various types of dyslipidemia (OR > 1, *p* value of IVW < 0.05) is as follows: Coprococcus2, Lachnospiraceae NK4A136 group, Lactobacillus, and Parabacteroides for HDL-C (Figure 1A), Parasutterella, Ruminococcus2, and Terrisporobacter for LDL-C (Figure 1B), Eubacterium coprostanoligenes group and Lactococcus for TC (Figure 1C), Coprobacter and Olsenella for TG (Figure 1D), Lactobacillus, Parabacteroides, and Ruminococcaceae UCG010 for APOA1 (Figure 1E), and Parasutterella and Terrisporobacter for APOB (Figure 1F). The results indicate that a higher prevalence of these bacterial species in the gastrointestinal tract is associated with elevated levels of certain lipids. Supplementary Materials S1 offers extensive details on the association between statistically significant gut microbiota genera and various types of lipid disorders in the MR analysis.

### 3.2. Sensitivity Analyses

**Figure 2:**
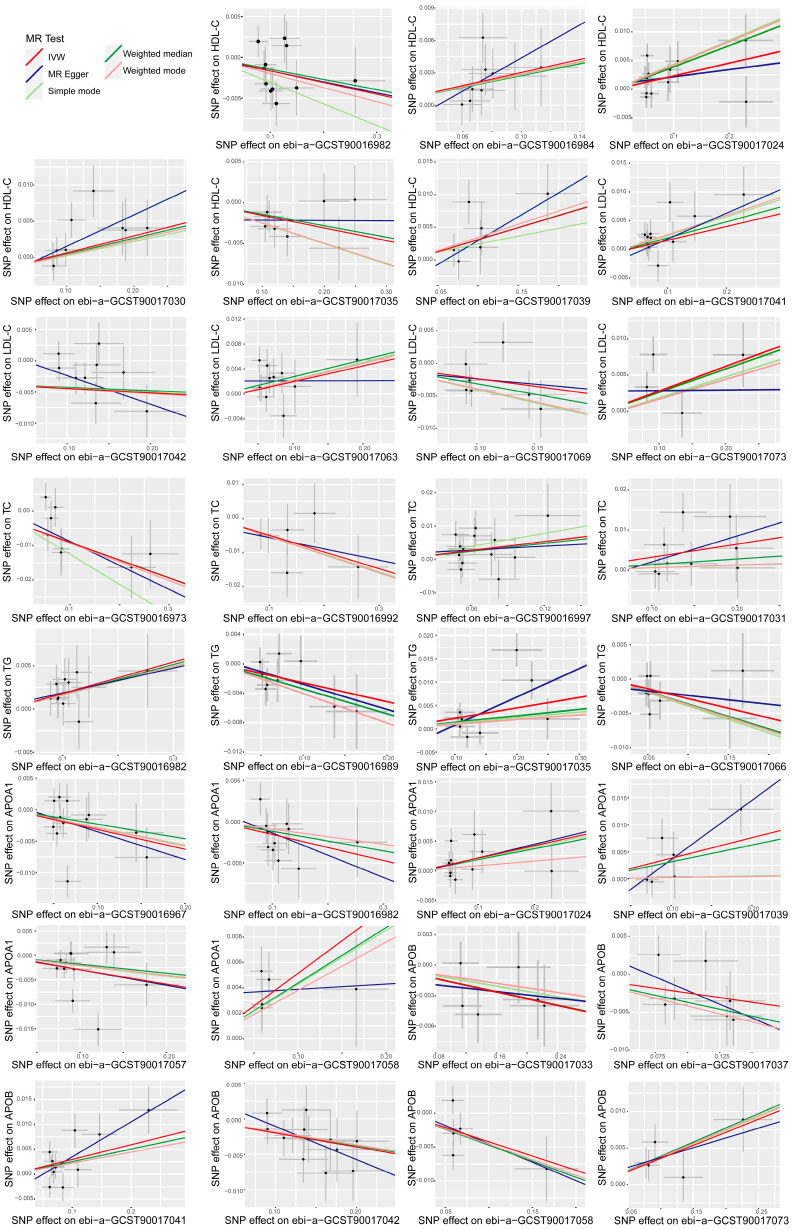
Scatter plot demonstrates a significant linear association between distinct gut microbiota genera and various forms of lipid metabolism within the human body, while no discernible heterogeneity of single nucleotide polymorphisms (SNPs) was observed. The black dots correspond to the single nucleotide polymorphisms (SNPs) employed in the Mendelian randomization analysis. The lines depict the linear fitting trends derived from various analysis methods employed in the Mendelian randomization analysis. The red line represents the fitting trend obtained through the inverse variance weighted (IVW) method, the dark blue line represents the fitting trend obtained through the MR-Egger method, the green line represents the fitting trend obtained through the weighted median method, the light red line represents the fitting trend obtained through the weighted mode method, and the light yellow green line represents the fitting trend obtained through the simple mode method. high-density lipoprotein cholesterol, HDL-C; low-density lipoprotein cholesterol, LDL-C; triglyceride, TG; total cholesterol, TC; apolipoprotein A1, APOA1; apolipoprotein B, APOB; SNP, single nucleotide polymorphisms.

Sensitivity tests were conducted using the MR Egger test to investigate the presence of horizontal pleiotropy among various gut microbiota genera associated with different types of dyslipidemia. The results revealed no significant evidence of horizontal pleiotropy, as indicated by *p* values exceeding 0.05 for the MR-Egger regression intercept approach (Table S2). However, there was significant heterogeneity (*p* < 0.05) in the causal associations between Olsenella and TG, Anaerotruncus, Ruminococcaceae UCG009, and APOA1, and the effect size for these associations was estimated using the random effect model of the IVW method, while a fixed effects model was employed to assess other causal effect sizes. The ultimate findings demonstrated that all effect values were statistically significant (*p* < 0.05, Table S2), thereby confirming the causal association between these gut microbiota genera and the regulation of lipid metabolism. Furthermore, the sensitivity analysis was performed using the leave-one-out method to assess the impact of individual SNPs on outcome estimation, and the findings consistently persisted (Supplementary Materials S2). The scatter plot, depicting the MR estimate of the effect of various gut microbiota genera on different dyslipidemia types, exhibited a clear linear trend (Figure 2). The funnel plot demonstrated minimal heterogeneity (Supplementary Materials S3). Collectively, these pieces of evidence strongly support the statistical robustness of the analysis results and the reliability of the conclusion.

**Figure 3:**
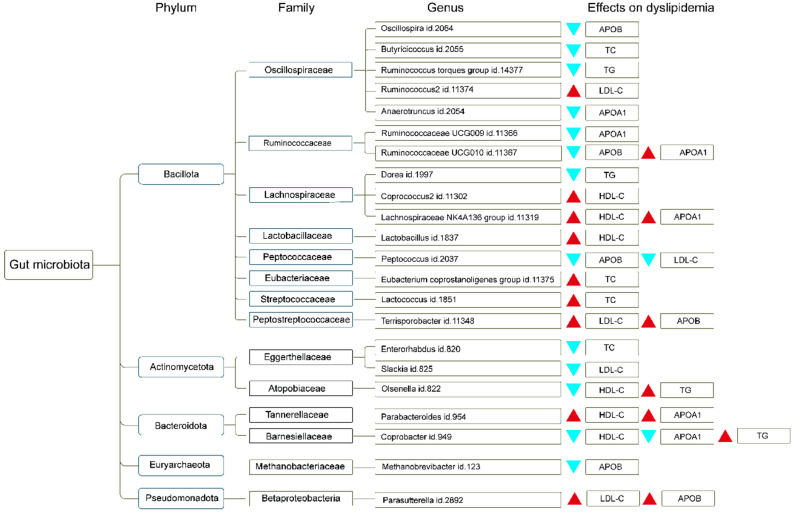
A comprehensive overview of the distribution of gut microbiota genera at the phylum and family levels, highlighting the subsequent influence on lipid levels. The red triangle represents the increasing effect, while the blue triangle represents the reducing effect. high-density lipoprotein cholesterol, HDL-C; low-density lipoprotein cholesterol, LDL-C; triglyceride, TG; total cholesterol, TC; apolipoprotein A1, APOA1; apolipoprotein B, APOB.

To enhance the understanding of the regulatory influence of gut microbiota genera on dyslipidemia, we summarized the phylum and family corresponding to different gut microbiota genera and their effects on different types of dyslipidemia, as shown in Figure 3.

## 4. Discussion

Dyslipidemia is a prevalent manifestation of metabolic disorders and has emerged as a significant global public health concern, posing a substantial threat to human well-being [93,94,95]. Nonetheless, the etiology of dyslipidemia remains intricate and inconclusive. The gut microbiota, being the largest microbiota within the human body [6,96], assumes a crucial function in various aspects such as nutrition metabolism, growth and development, immunity, and disease onset [16,97,98,99]. Despite the existing literature substantiating the association between gut microbiota and dyslipidemia [97], the presence of a causal link remains uncertain. To address this gap in knowledge, we employed MR analysis to investigate the potential causal associations between various gut microbiota genera implicated in the regulation of lipid metabolism. Our findings yielded enlightening evidence in this regard. The findings of this investigation primarily highlight two pivotal observations: Firstly, the two-sample MR analysis has revealed a distinct causal association between gut microbiota and dyslipidemia, thereby presenting novel evidence regarding the involvement of gut microbiota in the regulation of physiological processes. Secondly, the inconsistent effects of gut microbiota derived from various taxonomic ranks, including different phylum, families, and genera, on lipid metabolism further substantiate the widespread and comprehensive influence of gut microbiota on the regulation of bodily functions. In sum, these findings will provide valuable insights for enhancing our comprehension of the influence of gut microbiota on the physiological aspects of growth, development, and pathological states within the human body. 

Based on the observed distribution characteristics of bacterial phyla and families, our findings have identified the presence of up to 15 gut microbiota belonging to the Bacillota phylum and distributed across eight distinct families, which also exhibited the highest phylum distribution among the gut microbiota identified in our study. Among them, we observed the presence of 5 distinct types of gut microbiota (Oscillospira, Butyricicoccus, Ruminococcus torques group, Ruminococcus2, and Anaerotruncus genus) from the Oscillospiraceae family, each playing distinct roles in lipid regulation. Ruminococcus2 and Anaerotruncus had the potential to increase lipid levels in the body, whereas other bacteria, such as Oscillospira, Butyricicoccus, and the Ruminococcus torques group genus, demonstrated the ability to decrease lipid levels. Oscillospiraceae is a bacterial family classified within the phylum Bacillota, consisting of obligate anaerobes. Despite the variation in shapes among its members, including rod-shaped and cocci forms [100], the Oscillospira genus was recognized as a crucial type within the gut microbiota. Several studies have substantiated a significant positive correlation between Oscillospira and low fat, leanness, constipation, and overall human health [101,102]. However, it is imperative to acknowledge that this microorganism has yet to be successfully cultured in isolation, and its metabolic and biological characteristics remain largely unknown [103]. In the present study, we have identified a negative regulatory association between Oscillospira and APOB levels, aligning with prior research on the physiological mechanisms by which Oscillospira modulate bodily functions, such as lower body mass index (BMI) [102]. These cumulative findings further augmented the plausibility of Oscillospira as a prospective contender for forthcoming probiotic interventions. 

According to reports, there is a significant association between a decrease in Butyricicoccus and the occurrence of inflammatory bowel disease (IBD) [104]. IBD encompasses a group of inflammatory disorders of unknown origin, characterized by compromised immune system functioning in the intestines and metabolic irregularities [105,106]. In our study, we identified the significance of Butyricicoccus in the reduction in TC levels. This finding highlighted the potential regulatory function of Butyricicoccus in the body’s lipid metabolism and its association with disease processes related to lipid metabolism. Moreover, this evidence contributed to our existing knowledge regarding the involvement of gut microbiota in the pathogenesis of these diseases by modulating lipid metabolism. Our study has also identified two genera, namely Ruminococcus torques group and Ruminococcus2, belonging to the Oscillospiraceae family [107]. These genera exhibited distinct effects on dyslipidemia, with the Ruminococcus torques group reducing lipid levels and Ruminococcus2 evaluating lipid levels. Previous research has reported a lower abundance of the Ruminococcus genus in individuals with IBD [108], Parkinson’s disease [109], or Amyotrophic lateral sclerosis [110,111]. Furthermore, Ruminococcus gnavus has been associated with Crohn’s disease [112]. 

In relation to the Ruminococcaceae family, we have identified two gut microbiota genera that exhibit distinct effects on lipid levels in the body. Specifically, the Ruminococcaceae UCG009 genus appears to decrease APOA1 levels, while the Ruminococcaceae UCG010 genus appears to decrease APOB levels. The Ruminococcaceae family is known to play a role in energy metabolism, insulin signaling, and inflammatory processes. Moreover, an increase in the relative abundance of Ruminococcaceae has been found to increase the risk of gestational diabetes mellitus (GDM) development [113]. In a study utilizing mice as an experimental model, the authors observed that the Ruminococcaceae family exhibits a mitigating impact on the fibrosis of nonalcoholic fatty liver disease (NAFLD) [114] and modulates hepatic fat content and lipid species composition [115].

The genera of gut microbiota belonging to the families Lachnospiraceae, Lactobacillaceae, and Peptococcaceae within the Bacillota phylum have been found to have significant positive effects on lipid levels in the human body. Notably, the genera Dorea, Coprococcus2, and Lachnospiraceae NK4A136 group from the Lachnospiraceae family, Lactobacillus from the Lactobacillaceae family, and Peptococcus from the Peptococcaceae family have demonstrated a dual role in regulating lipid metabolism. These gut microbiota have the ability to reduce harmful lipids (APOB and LDL-C) while also promoting the evaluation of beneficial lipids (HDL-C and APOA1) in the body. Lachnospiraceae, a prominent taxon in the human gut microbiota, has been found to potentially mitigate colon cancer in humans using the production of butyric acid [116,117,118]. Additionally, it was reported that the reduction in Lachnospiraceae abundance has been associated with Chronic Spontaneous Urticaria [119], sleep deprivation [120], and obesity [121]. As is known to all, the Lactobacillus genus plays a significant role in the microbiota of both humans and animals, particularly in various body sites such as the digestive and female genital systems [122]. Lactobacillus demonstrates a mutualistic symbiosis with the human body, wherein it serves to safeguard the host against potential pathogenic incursions while the host reciprocally offers a nutrient source [123,124]. A randomized controlled trial (RCT) has discerned that Lactobacillus exerts a positive influence on glucose metabolism in pregnant women who are overweight or obese [125]. The results of our research demonstrate that Lactobacillus exerts positive effects on correcting abnormal lipid metabolism levels. These findings further underscore the essential role of Lactobacillus as indispensable probiotics in the physiological mechanisms of the human body. The Peptococcus genus, a Gram-positive bacterium genus within the family Peptococcaceae, is frequently observed in the human microbiome, specifically in the gut flora, as well as in the oral cavity, and upper respiratory tract. Our research findings provide additional evidence supporting a significant correlation between the Peptococcus genus and the decrease in LDL-C and APOB levels within the body, indicating a potential contribution to the the amelioration of dyslipidemia.

In addition, our investigation revealed that several families within the Bacillota phylum, such as the Eubacterium coprostanoligenes group from the Eubacteriaceae family, Lactococcus from the Streptococcaceae family, and Terrisporobacter from the Peptostreptococcaceae family, exert a noteworthy influence on the augmentation of lipid levels in the human organism. Significantly, these gut microbiota species demonstrated substantial effects on TC and LDL-C levels. The significance of this particular family resides in its ability to generate diverse strains that produce short-chain fatty acids, particularly butyric acid. These short-chain fatty acids are widely acknowledged for their pivotal functions in maintaining human health, including their function as specialized nutrients and energy sources for the intestinal epithelium, preserving the integrity of the intestinal mucosal barrier, reducing inflammation levels in humans, and enhancing gastrointestinal motility [126,127]. Lactococcus, a beneficial microbiota, is commonly utilized in the dairy industry for the production of fermented dairy products, such as cheeses. However, our study has substantiated a positive causal association between Lactococcus and TC levels, thereby implying that individuals with elevated blood lipid levels should avoid consuming cheese products. Terriporobacter, a member of the Peptostrectococcaceae family, is currently under investigation for its distinctive attributes and biological mechanisms. Our research findings suggest that this particular gut microbiota has the capability to elevate LDL-C and APOB levels, thereby categorizing it as a potentially detrimental microbiota.

In addition, our findings reveal the presence of additional phyla in the observed data, including three gut microbiota belonging to the Actinomycetota phylum, which are distributed among two families, and two gut microbiota belonging to the Bacteroidota phylum are distributed across two families. Moreover, within the Euryarchaeota and Pseudomonadota phylum, two distinct gut microbiota genera are identified, each belonging to their respective autonomous families. The Actinomycetota genus is prevalent in the microbiome of human infants [128] and is known for its production of bioactive metabolites with medicinal value [129]. Our study reveals a robust causal association between Eggerthellaceae and the reduction in TC and LDL-C levels in the human body. Conversely, the presence of Atopobiaceae bacteria is associated with elevated blood lipid levels, resulting in increased TG levels and decreased HDL-C levels. In a similar manner, two distinct families of gut microbiota, Tannellaceae, and Barnesiellaceae, which are affiliated with the Bacteroidota phylum, have shown inconsistent impacts on the regulation of lipid metabolism. Specifically, Tannellaceae bacteria have demonstrated the capacity to increase levels of HDL-C and APOA1, potentially mitigating the elevation of blood lipid levels. Conversely, the presence of Barnesiellaceae has been observed to decrease HDL-C and APOA1 levels while simultaneously increasing TG levels. The Metanobacteriaceae family, classified within the Euryarchaeota phylum, has been identified as a pathogenic microorganism. Our study findings indicate that this particular gut microbiota exerts a suppressive impact on APOB levels. Additionally, the presence of the Betaproteobacteria family from the Pseudomonas phylum exhibits a notable positive causal association with increased levels of LDL-C and APOB. This suggests a distinct propensity of this bacterial family to stimulate elevated lipid levels in the human body.

In conclusion, the influence of gut microbiota on lipid metabolism varies depending on the specific types of gut microbiota. Our study demonstrates that the predominant phylum of gut microbiota in humans also encompasses the most diverse microbial group responsible for regulating lipid metabolism. The families Lachnospiraceae and Lactobacillaceae are of notable importance and should be recognized as crucial microbiota in ameliorating dyslipidemia within the human body. Furthermore, it is imperative to recognize that individuals with hyperlipidemia should abstain from consuming cheese. Our research findings elucidate the wide-ranging and ubiquitous influence of gut microbiota on the regulation of lipid metabolism levels, thereby enhancing our comprehension of the interplay between gut microbiota and diseases associated with dyslipidemia. These results provide novel evidence that contributes to a more comprehensive understanding of how gut microbiota modulates bodily functions and metabolism.

Our study possesses several notable strengths, such as the implementation of the MR approach, which effectively mitigates certain confounding factors frequently encountered in epidemiological studies. Additionally, we have employed a homogenous population, thereby reducing the inherent heterogeneity often encountered when individuals from diverse ancestral backgrounds are included in genetic studies. Stratified analyses were employed to assess the causal associations between various genera of gut microbiota and distinct dyslipidemia types. Additionally, sensitivity analysis was conducted on the subgroup analysis outcomes, yielding statistically robust results. Nevertheless, it is important to note that the inclusion of exclusively European individuals in our analyses may limit the generalizability of these findings to other ancestral populations.

## 5. Conclusions

Our findings demonstrate a definitive causal link between gut microbiota and dyslipidemia within the human organism. Notably, the Bacillota phylum emerges as the most influential regulator of body lipid levels. The families Lachnospiraceae and Lactobacillaceae assume a noteworthy role in ameliorating lipid metabolism abnormalities and should be recognized as crucial gut microbiota in this process. Additionally, it is recommended that individuals with hyperlipidemia are advised to exercise caution when consuming cheese, as the prevalence of Lactococcus in this food item may potentially exert detrimental effects on their lipid profile.

## Data Availability

All data used in this study are available in the public repository. The code involved in the data analysis process can be obtained by contacting the corresponding author.

## Supplementary Materials

The following supporting information can be downloaded at: https://www.mdpi.com/article/10.3390/nu15204445/s1. Supplementary Materials S1 provides SNP details between gut microbiota and different types of dyslipidemia. The result from each gut microbiota is represented using a separate “CSV” format table. Supplementary Materials S2 provides the plots of Leaveoneout analysis between gut microbiota and different types of dyslipidemia. The result from each gut microbiota is represented using a separate “pdf” format figure. Supplementary Materials S3 provides the funnel plots of sensitivity analysis between gut microbiota and different types of dyslipidemia. The result from each type of dyslipidemia is represented using a separate “pdf” format figure.
