# Supplementary material for: Causal Associations between Gut Microbiota and Different Types of Dyslipidemia: A Two-Sample Mendelian Randomization Study
Source: Nutrients. 2023 Oct 20;15(20):4445. doi: 10.3390/nu15204445 (PMC10609956; doi:10.3390/nu15204445)
Supplement: Supplementary file 1 [file nutrients-15-04445-s001.zip › nutrients-2637637-SI.pdf]

nutrients MDPI

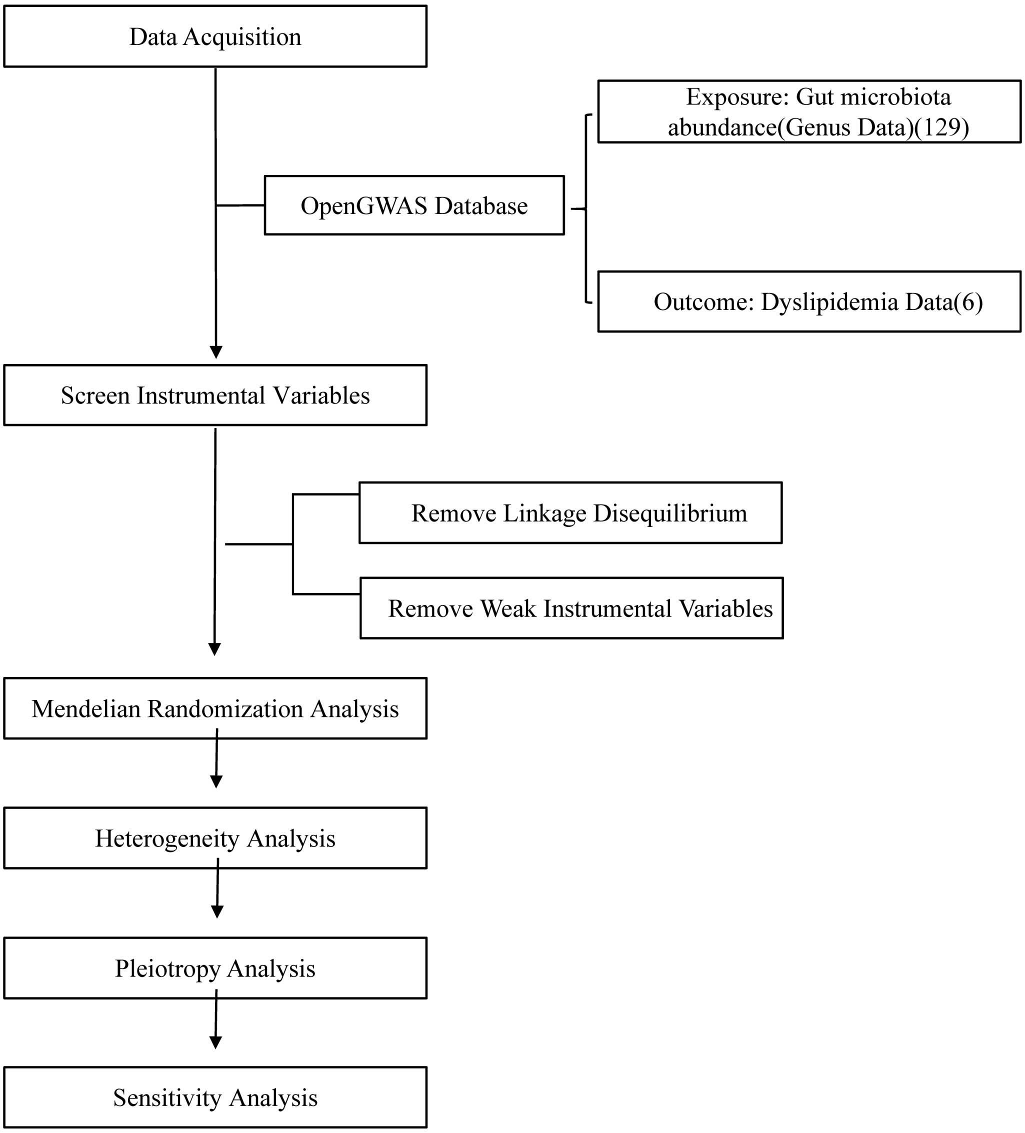

Figure S1. Flowchart of the study design.

Table S1. Comprehensive information pertaining to the dataset sourced from the GWAS database, which was utilized in the present study.

GWAS ID	Year	Trait	Population	Sex	Sample size	Number of SNPs	Author
EBI-A-GCST90016959—7087	2021	Gut microbiota abundance (Genus)	European	Males and Females	14306/Genus	5279880/Genus	Kurilshikov A
ieu-b-107	2020	apolipoprotein A-I	European	Males and Females	393,193	12,321,875	Richardson, Tom
ieu-b-108	2020	apolipoprotein B	European	Males and Females	439,214	12,321,875	Richardson, Tom
ieu-b-109	2020	HDL cholesterol	European	Males and Females	403,943	12,321,875	Richardson, Tom
ieu-b-110	2020	LDL cholesterol	European	Males and Females	440,546	12,321,875	Richardson, Tom
ieu-b-111	2020	Triglycerides	European	Males and Females	441,016	12,321,875	Richardson, Tom
met-d-Total_C	2020	Total cholesterol	European	Males and Females	115,078	12,321,875	Borges CM

Table S2. Sensitivity analysis for the significant results via mendelian randomization analysis. GM, gut microbiota, Pleio.p, p value of pleiotropy; Heter.p, p value of heterogeneity; Effect.p, p value of effect via inverse-variance weighted method. *, random effect model for inverse-variance weighted method.

Outcome	Exposure.id	GM genus	Pleio.p	Heter.p	Effect.p
HDL-C					
	ebi-a-GCST90016982	Coprobacter id.949	9.71E-01	2.63E-01	2.67E-02
	ebi-a-GCST90016984	Coprococcus2 id.11302	4.85E-01	7.47E-01	3.85E-03
	ebi-a-GCST90017024	Lachnospiraceae NK4A136 group id.11319	6.01E-01	2.22E-01	1.61E-02
	ebi-a-GCST90017030	Lactobacillus id.1837	2.79E-01	4.94E-01	6.99E-03
	ebi-a-GCST90017035	Olsenella id.822	4.76E-01	8.70E-01	9.34E-03
	ebi-a-GCST90017039	Parabacteroides id.954	4.13E-01	4.18E-01	2.58E-03
LDL-C					
	ebi-a-GCST90017041	Parasutterella id.2892	3.98E-01	7.48E-01	1.82E-03
	ebi-a-GCST90017042	Peptococcus id.2037	3.11E-01	5.07E-01	4.08E-02
	ebi-a-GCST90017063	Ruminococcus2 id.11374	4.50E-01	5.07E-01	1.48E-02
	ebi-a-GCST90017069	Slackia id.825	9.02E-01	2.21E-01	1.36E-02
	ebi-a-GCST90017073	Terrisporobacter id.11348	5.66E-01	1.05E-01	1.85E-03
TC					
	ebi-a-GCST90016973	Butyricicoccus id.2055	6.11E-01	3.55E-01	6.04E-03
	ebi-a-GCST90016992	Enterorhabdus id.820	7.60E-01	4.97E-01	1.29E-02
	ebi-a-GCST90016997	Eubacterium coprostanoligenes group id.11375	7.72E-01	5.14E-01	4.53E-02
	ebi-a-GCST90017031	Lactococcus id.1851	6.15E-01	3.21E-01	1.18E-02
TG					
	ebi-a-GCST90016982	Coprobacter id.949	8.70E-01	9.64E-01	1.12E-02
	ebi-a-GCST90016989	Dorea id.1997	7.92E-01	7.52E-01	3.84E-02
	ebi-a-GCST90017035	Olsenella id.822	2.89E-01	1.27E-02	2.63E-02*
	ebi-a-GCST90017066	Ruminococcus torques group id.14377	6.21E-01	4.40E-01	3.32E-02
APOA1					
	ebi-a-GCST90016967	Anaerotruncus id.2054	7.93E-01	1.37E-02	4.29E-02*
	ebi-a-GCST90016982	Coprobacter id.949	6.32E-01	5.59E-01	2.54E-02
	ebi-a-GCST90017024	Lachnospiraceae NK4A136 group id.11319	8.93E-01	4.36E-01	2.33E-02
	ebi-a-GCST90017039	Parabacteroides id.954	1.54E-01	5.12E-01	7.86E-03
	ebi-a-GCST90017057	Ruminococcaceae UCG009 id.11366	9.67E-01	2.29E-03	1.38E-02*
	ebi-a-GCST90017058	Ruminococcaceae UCG010 id.11367	3.34E-01	6.74E-01	2.99E-04
APOB					
	ebi-a-GCST90017033	Methanobrevibacter id.123	7.77E-01	5.03E-01	3.19E-02
	ebi-a-GCST90017037	Oscillospira id.2064	3.89E-01	4.05E-01	3.38E-02
	ebi-a-GCST90017041	Parasutterella id.2892	1.27E-01	3.30E-01	6.23E-04
	ebi-a-GCST90017042	Peptococcus id.2037	2.96E-01	9.42E-01	2.27E-03
	ebi-a-GCST90017058	Ruminococcaceae UCG010 id.11367	8.75E-01	5.23E-02	5.08E-03
	ebi-a-GCST90017073	Terrisporobacter id.11348	7.69E-01	5.16E-01	1.05E-03
