# Supplementary material for: Causal Associations between Gut Microbiota and Different Types of Dyslipidemia: A Two-Sample Mendelian Randomization Study
Source: Nutrients. 2023 Oct 20;15(20):4445. doi: 10.3390/nu15204445 (PMC10609956; doi:10.3390/nu15204445)
Supplement: Supplementary file 1 [file nutrients-15-04445-s001.zip › Supplementary materials 2/Leaveoneout plot for gut microbiota on APOA1/Leaveoneout plot for ebi-a-GCST90017058 on APOA1.pdf]

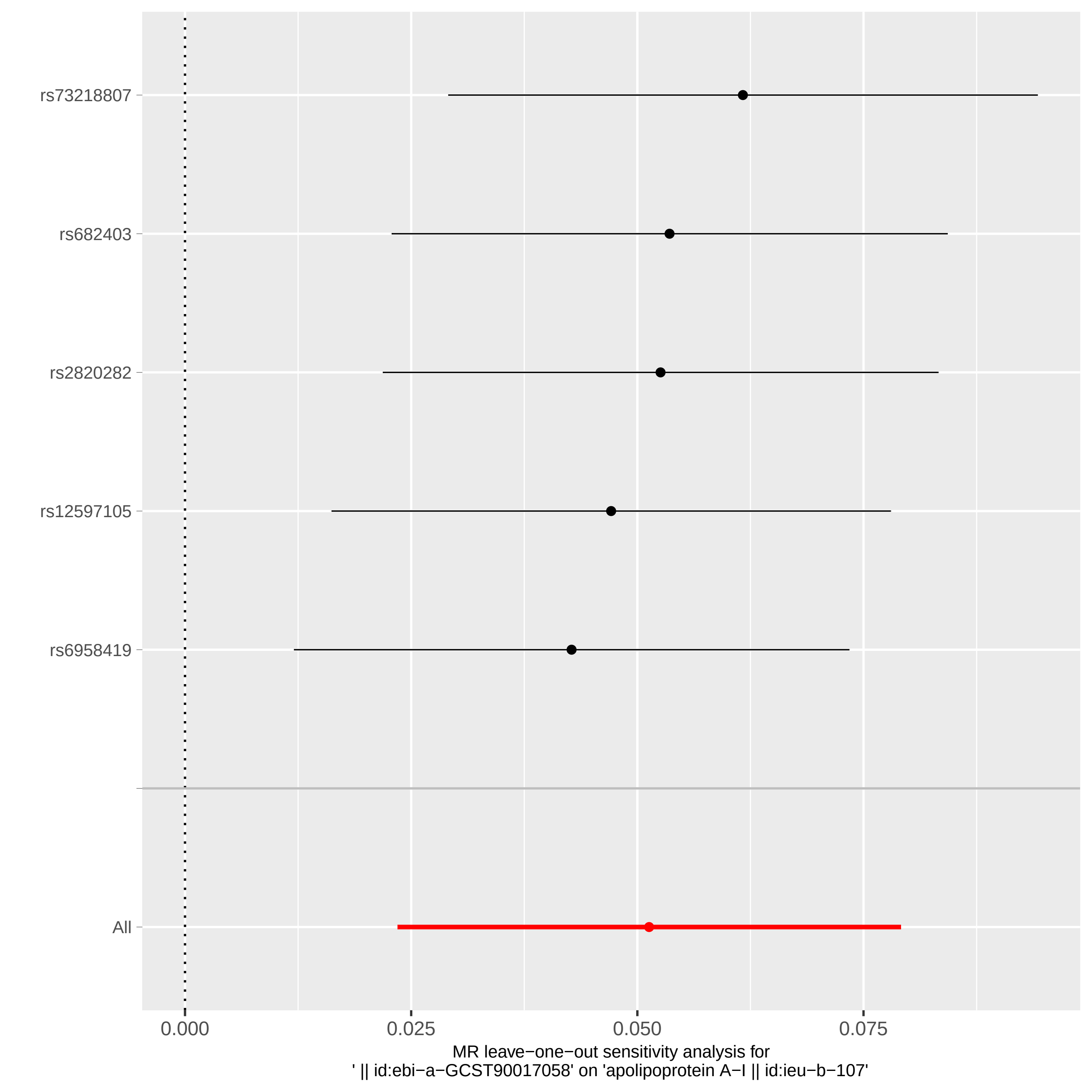

rs73218807
rs682403
rs2820282
rs12597105
rs6958419
All
0.000
0.025
0.050
0.075
MR leave−one−out sensitivity analysis for
' || id:ebi−a−GCST90017058' on 'apolipoprotein A−I || id:ieu−b−107'
