# Supplementary material for: Causal Associations between Gut Microbiota and Different Types of Dyslipidemia: A Two-Sample Mendelian Randomization Study
Source: Nutrients. 2023 Oct 20;15(20):4445. doi: 10.3390/nu15204445 (PMC10609956; doi:10.3390/nu15204445)
Supplement: Supplementary file 1 [file nutrients-15-04445-s001.zip › Supplementary materials 2/Leaveoneout plot for gut microbiota on APOB/Leaveoneout plot for ebi-a-GCST90017033 on APOB.pdf]

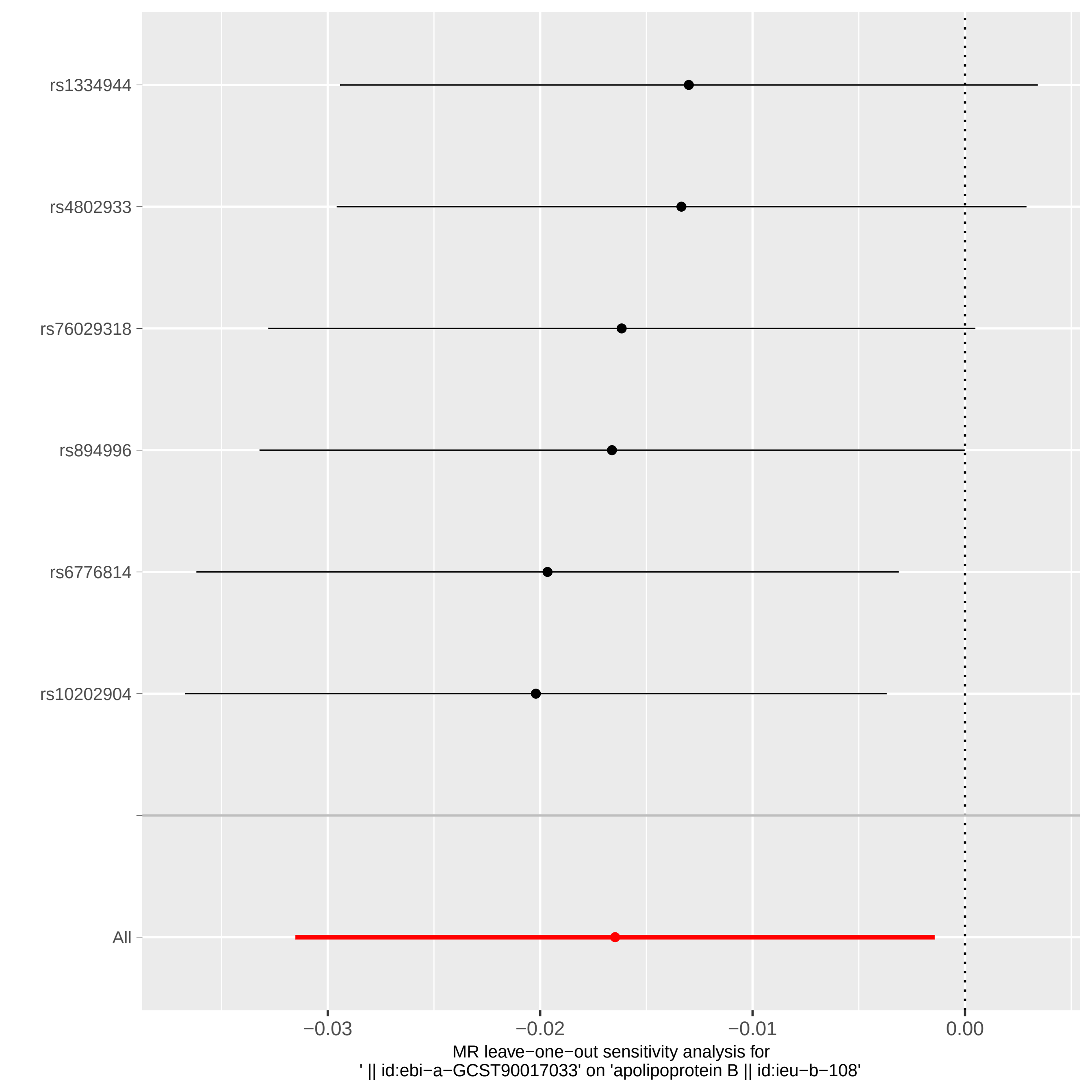
rs1334944
rs4802933
rs76029318
rs894996
rs6776814
rs10202904
All
−0.03
−0.02
−0.01
0.00
MR leave−one−out sensitivity analysis for
' || id:ebi−a−GCST90017033' on 'apolipoprotein B || id:ieu−b−108'
