# Supplementary material for: Causal Associations between Gut Microbiota and Different Types of Dyslipidemia: A Two-Sample Mendelian Randomization Study
Source: Nutrients. 2023 Oct 20;15(20):4445. doi: 10.3390/nu15204445 (PMC10609956; doi:10.3390/nu15204445)
Supplement: Supplementary file 1 [file nutrients-15-04445-s001.zip › Supplementary materials 2/Leaveoneout plot for gut microbiota on APOB/Leaveoneout plot for ebi-a-GCST90017073 on APOB.pdf]

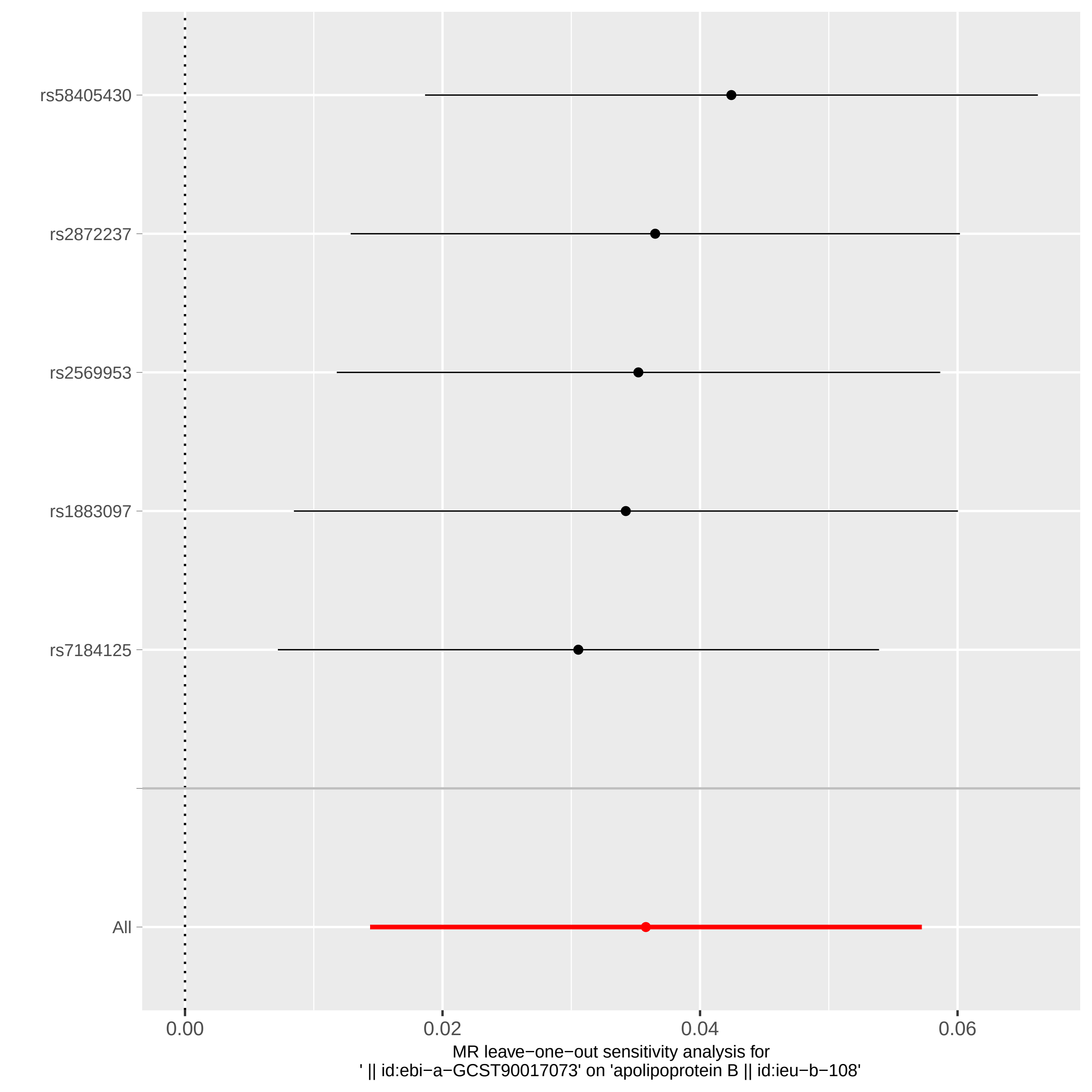
rs58405430
rs2872237
rs2569953
rs1883097
rs7184125
All
0.00
0.02
0.04
0.06
MR leave−one−out sensitivity analysis for
' || id:ebi−a−GCST90017073' on 'apolipoprotein B || id:ieu−b−108'
