# Supplementary figures and images for: Causal Associations between Gut Microbiota and Different Types of Dyslipidemia: A Two-Sample Mendelian Randomization Study
Source: Nutrients. 2023 Oct 20;15(20):4445. doi: 10.3390/nu15204445 (PMC10609956; doi:10.3390/nu15204445)

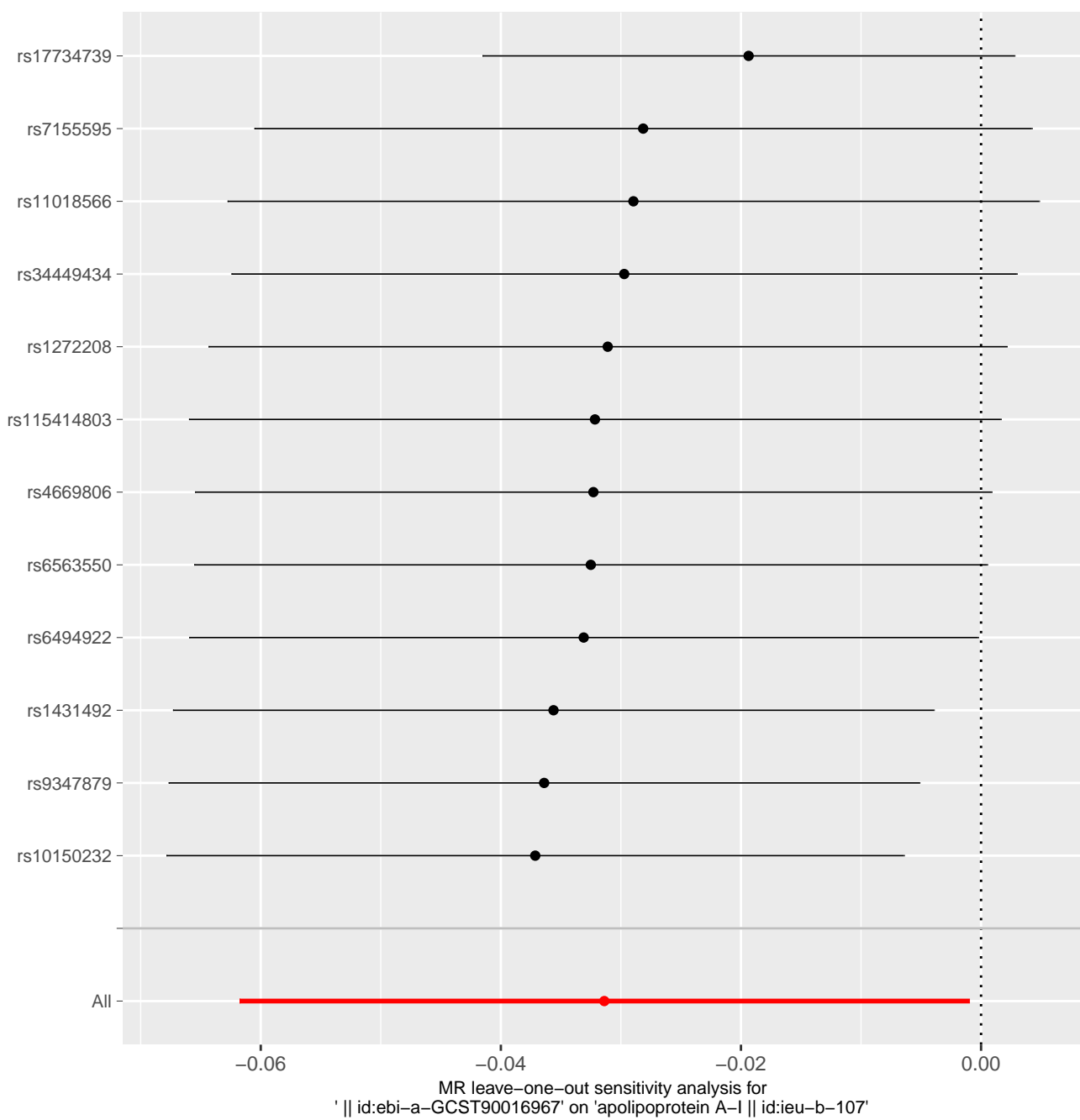

Supplement: Supplementary file 1 [file nutrients-15-04445-s001.zip › Supplementary materials 2/Leaveoneout plot for gut microbiota on APOA1/Leaveoneout plot for ebi-a-GCST90016967 on APOA1.pdf]

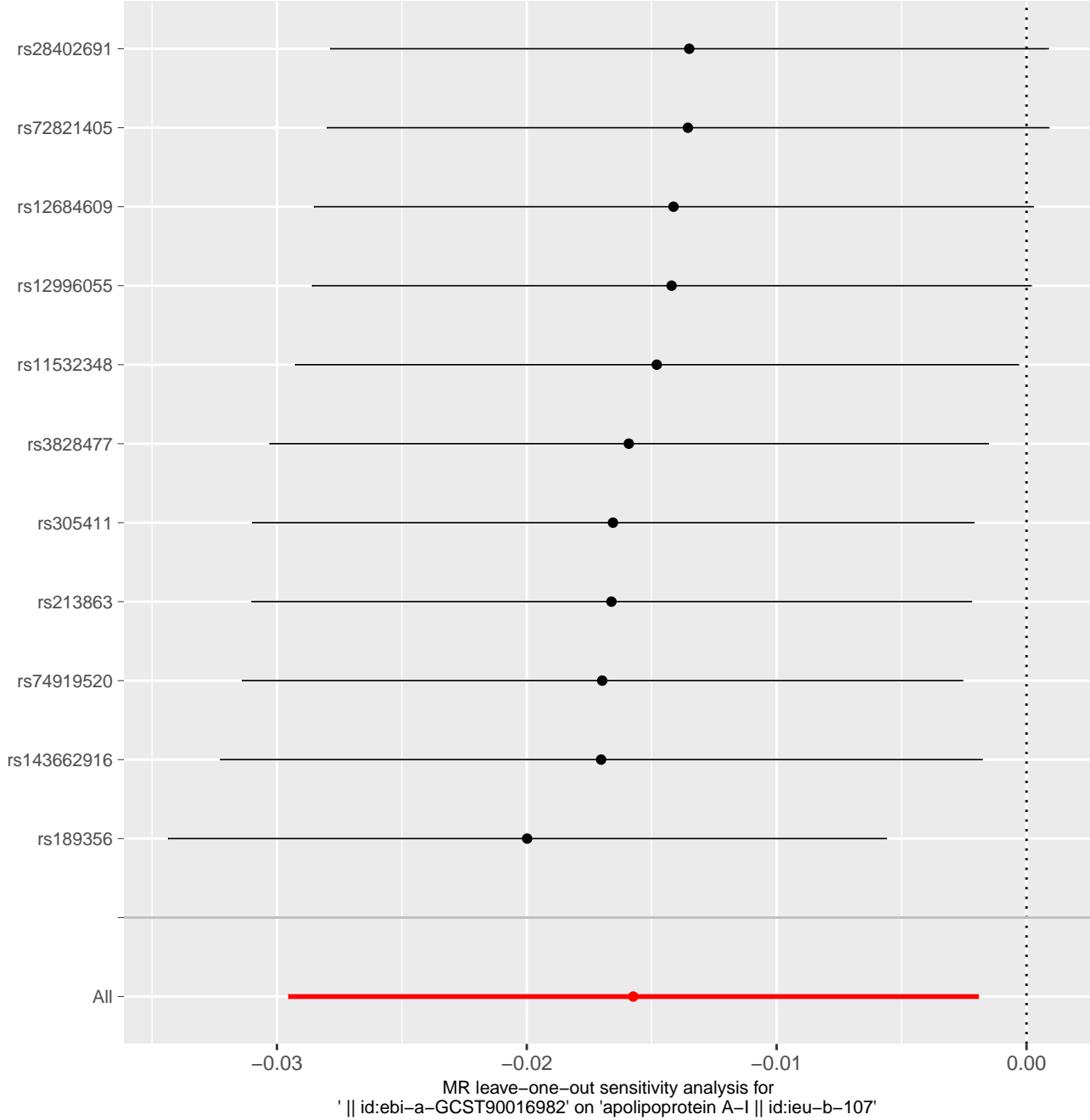

Supplement: Supplementary file 1 [file nutrients-15-04445-s001.zip › Supplementary materials 2/Leaveoneout plot for gut microbiota on APOA1/Leaveoneout plot for ebi-a-GCST90016982 on APOA1.pdf]

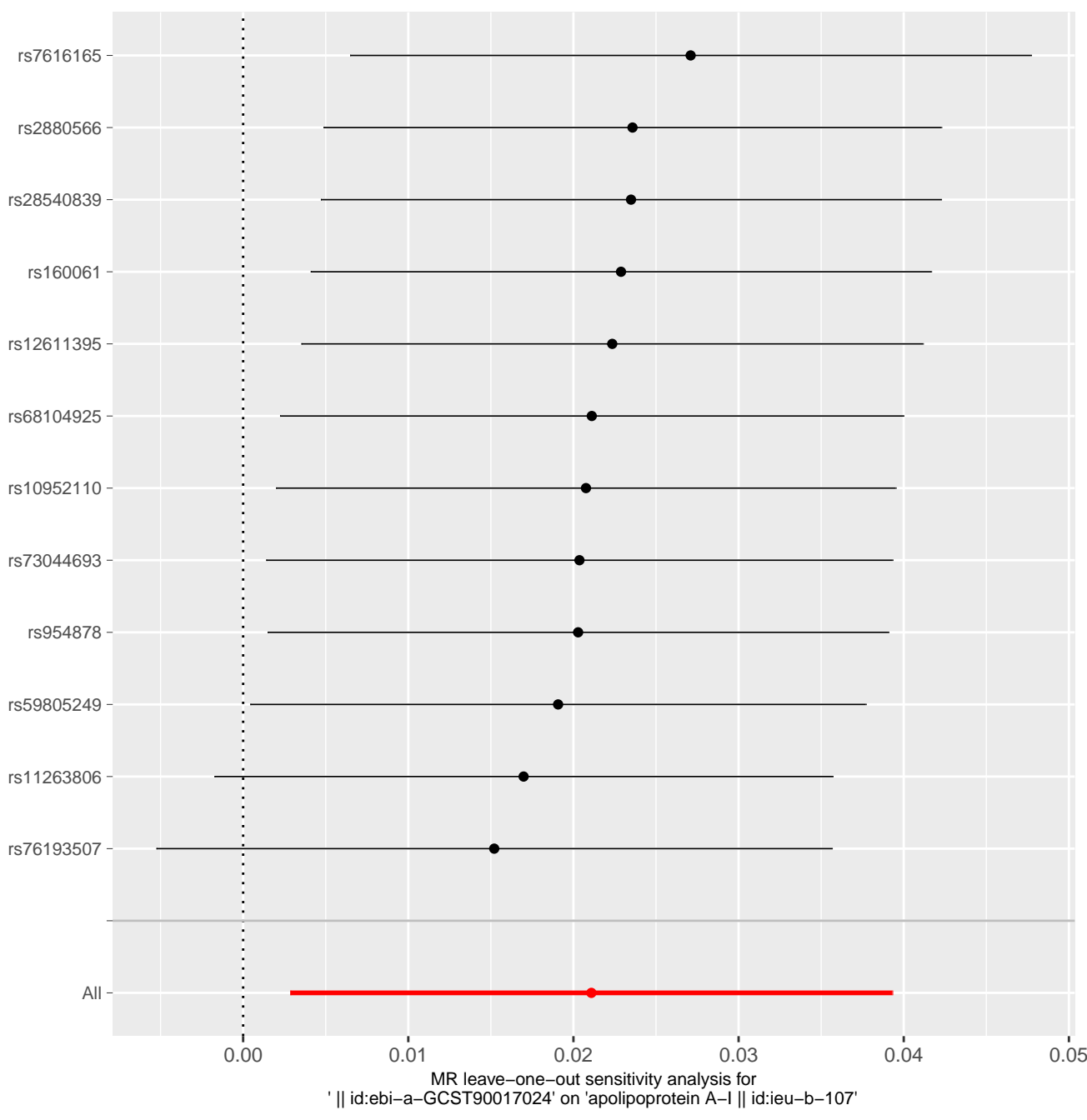

Supplement: Supplementary file 1 [file nutrients-15-04445-s001.zip › Supplementary materials 2/Leaveoneout plot for gut microbiota on APOA1/Leaveoneout plot for ebi-a-GCST90017024 on APOA1.pdf]

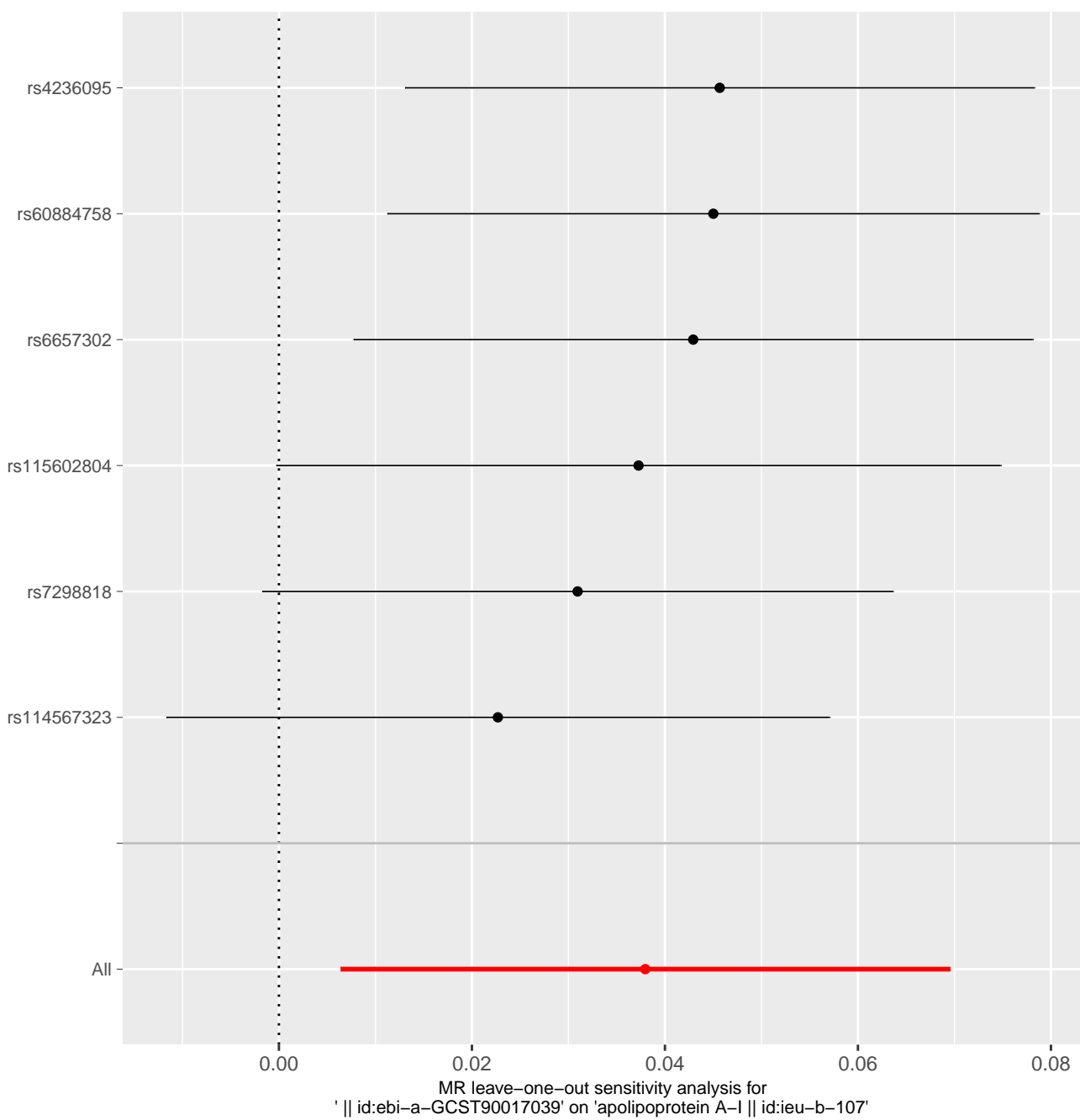

Supplement: Supplementary file 1 [file nutrients-15-04445-s001.zip › Supplementary materials 2/Leaveoneout plot for gut microbiota on APOA1/Leaveoneout plot for ebi-a-GCST90017039 on APOA1.pdf]

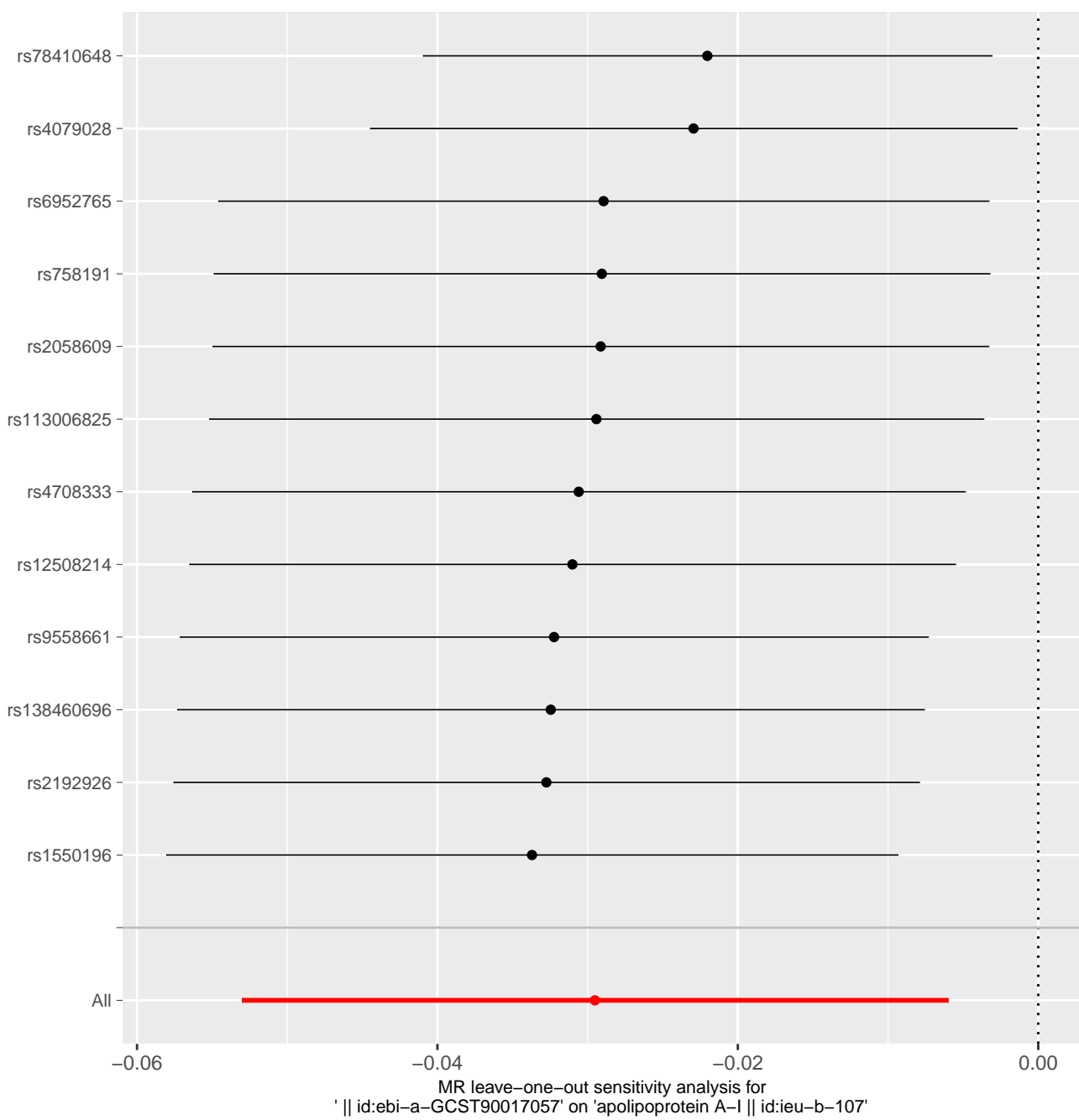

Supplement: Supplementary file 1 [file nutrients-15-04445-s001.zip › Supplementary materials 2/Leaveoneout plot for gut microbiota on APOA1/Leaveoneout plot for ebi-a-GCST90017057 on APOA1.pdf]

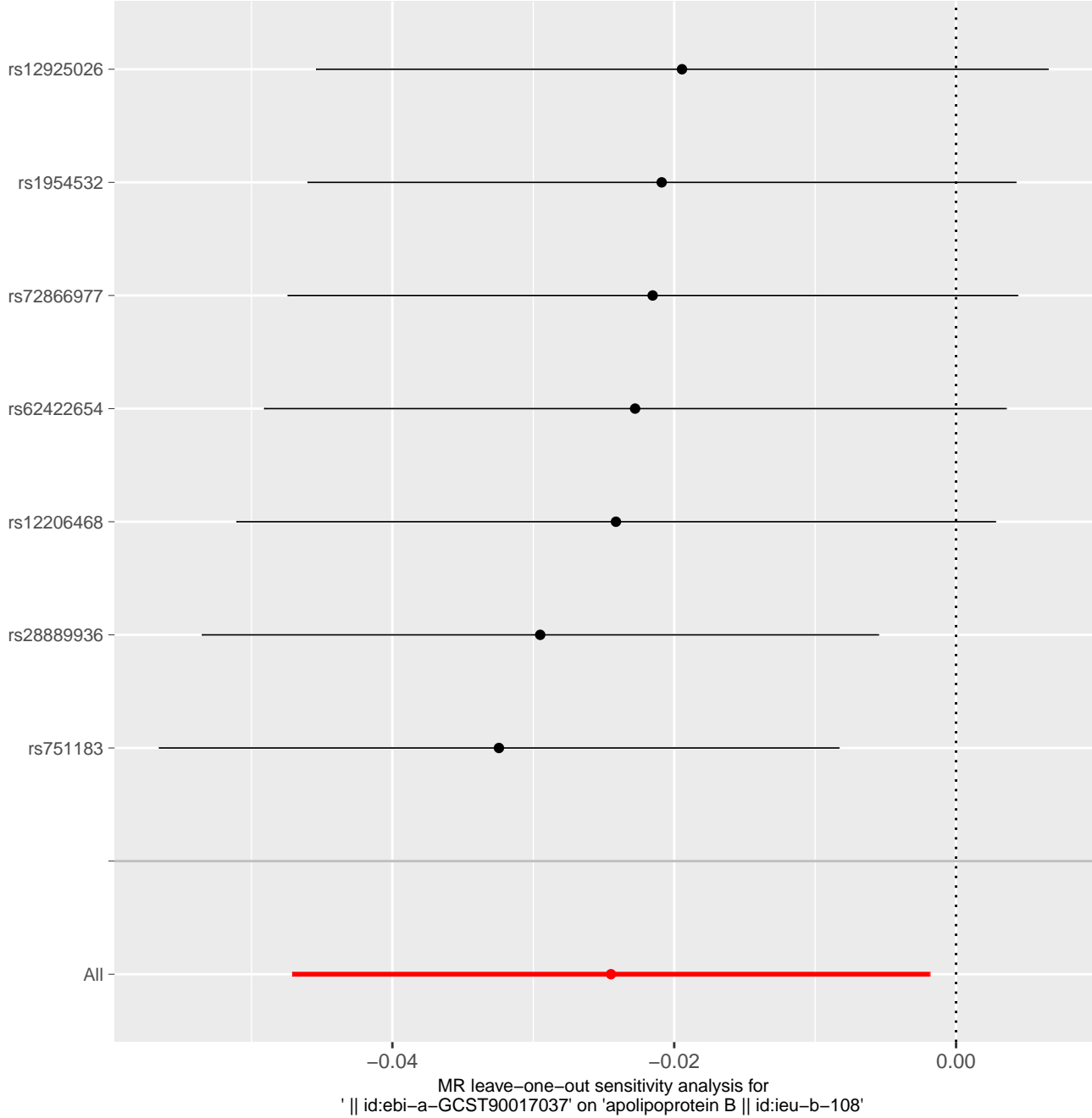

Supplement: Supplementary file 1 [file nutrients-15-04445-s001.zip › Supplementary materials 2/Leaveoneout plot for gut microbiota on APOB/Leaveoneout plot for ebi-a-GCST90017037 on APOB.pdf]

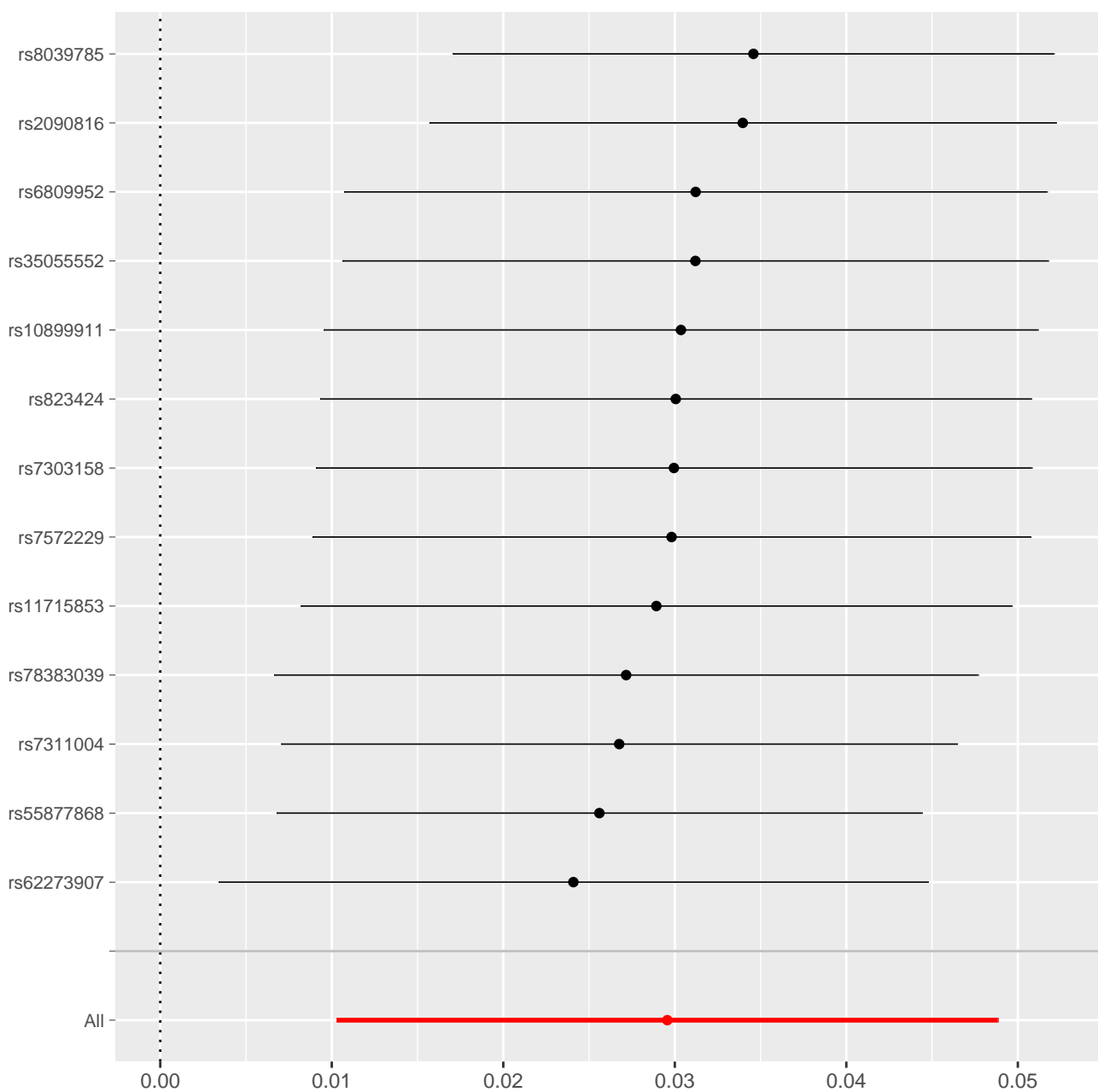

Supplement: Supplementary file 1 [file nutrients-15-04445-s001.zip › Supplementary materials 2/Leaveoneout plot for gut microbiota on APOB/Leaveoneout plot for ebi-a-GCST90017041 on APOB.pdf]

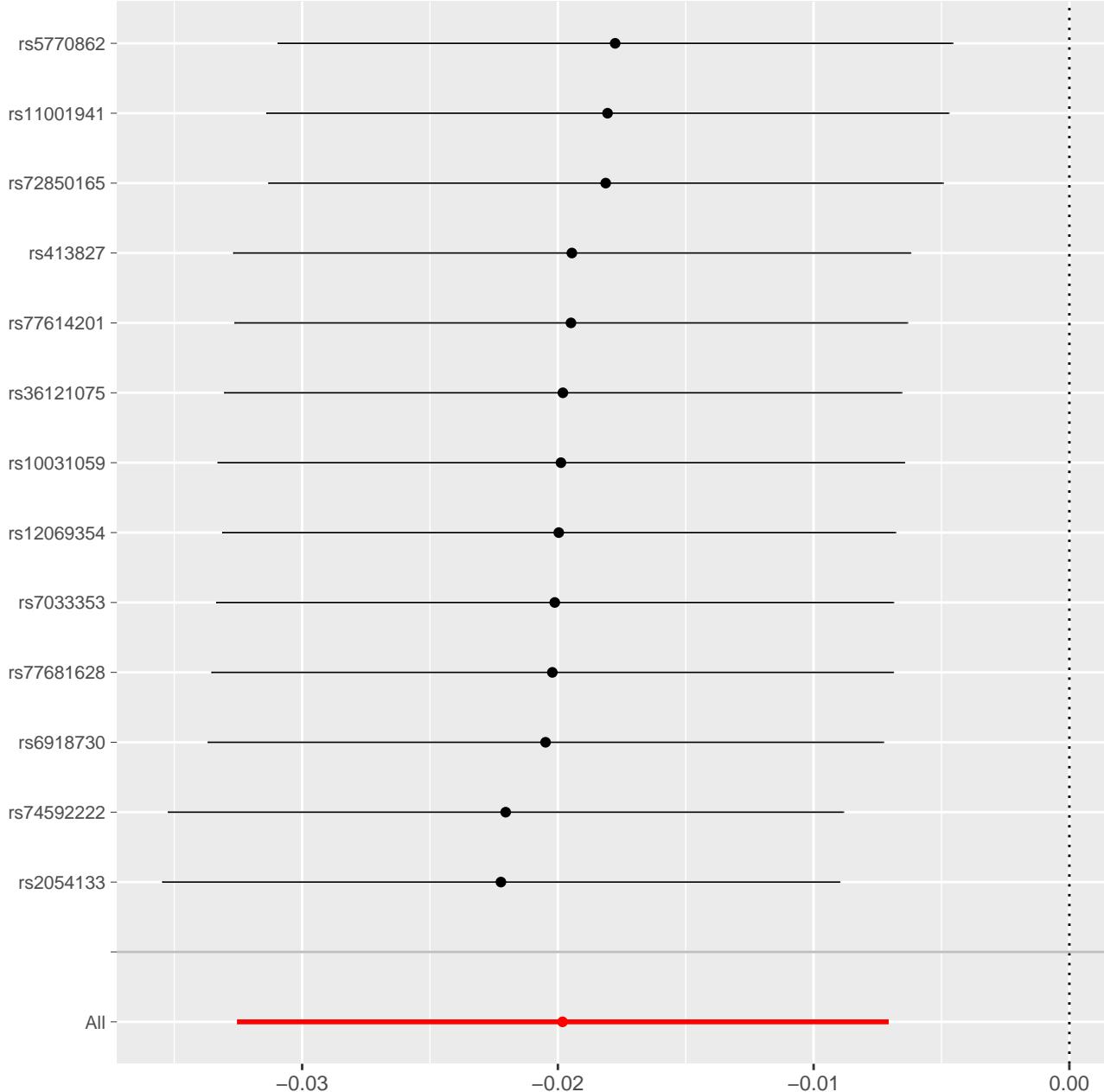

Supplement: Supplementary file 1 [file nutrients-15-04445-s001.zip › Supplementary materials 2/Leaveoneout plot for gut microbiota on APOB/Leaveoneout plot for ebi-a-GCST90017042 on APOB.pdf]

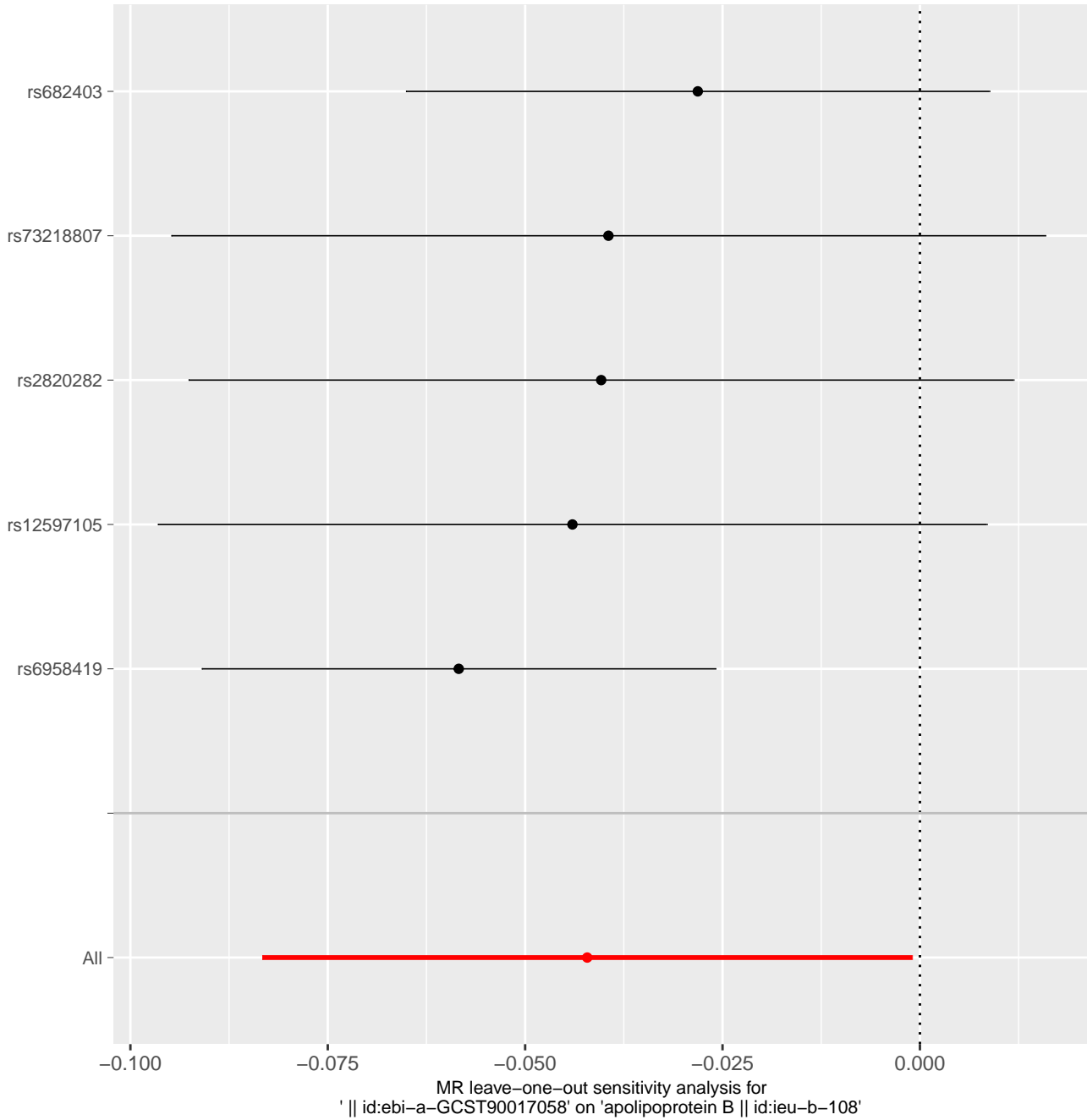

Supplement: Supplementary file 1 [file nutrients-15-04445-s001.zip › Supplementary materials 2/Leaveoneout plot for gut microbiota on APOB/Leaveoneout plot for ebi-a-GCST90017058 on APOB.pdf]

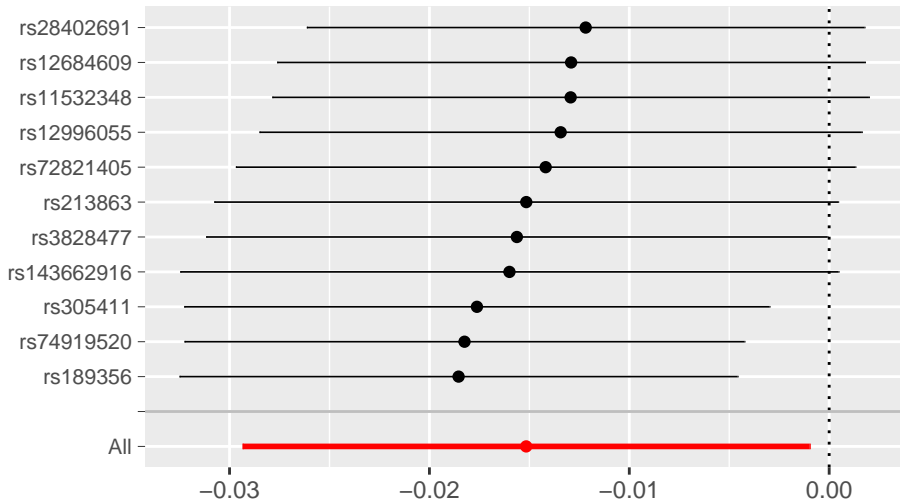

MR leave-one-out sensitivity analysis for  
' || id:ebi-a-GCST90016982' on 'HDL cholesterol || id:ieu-b-109'

Supplement: Supplementary file 1 [file nutrients-15-04445-s001.zip › Supplementary materials 2/Leaveoneout plot for gut microbiota on HDL-C/Leaveoneout plot for ebi-a-GCST90016982 on HDL-C.pdf]

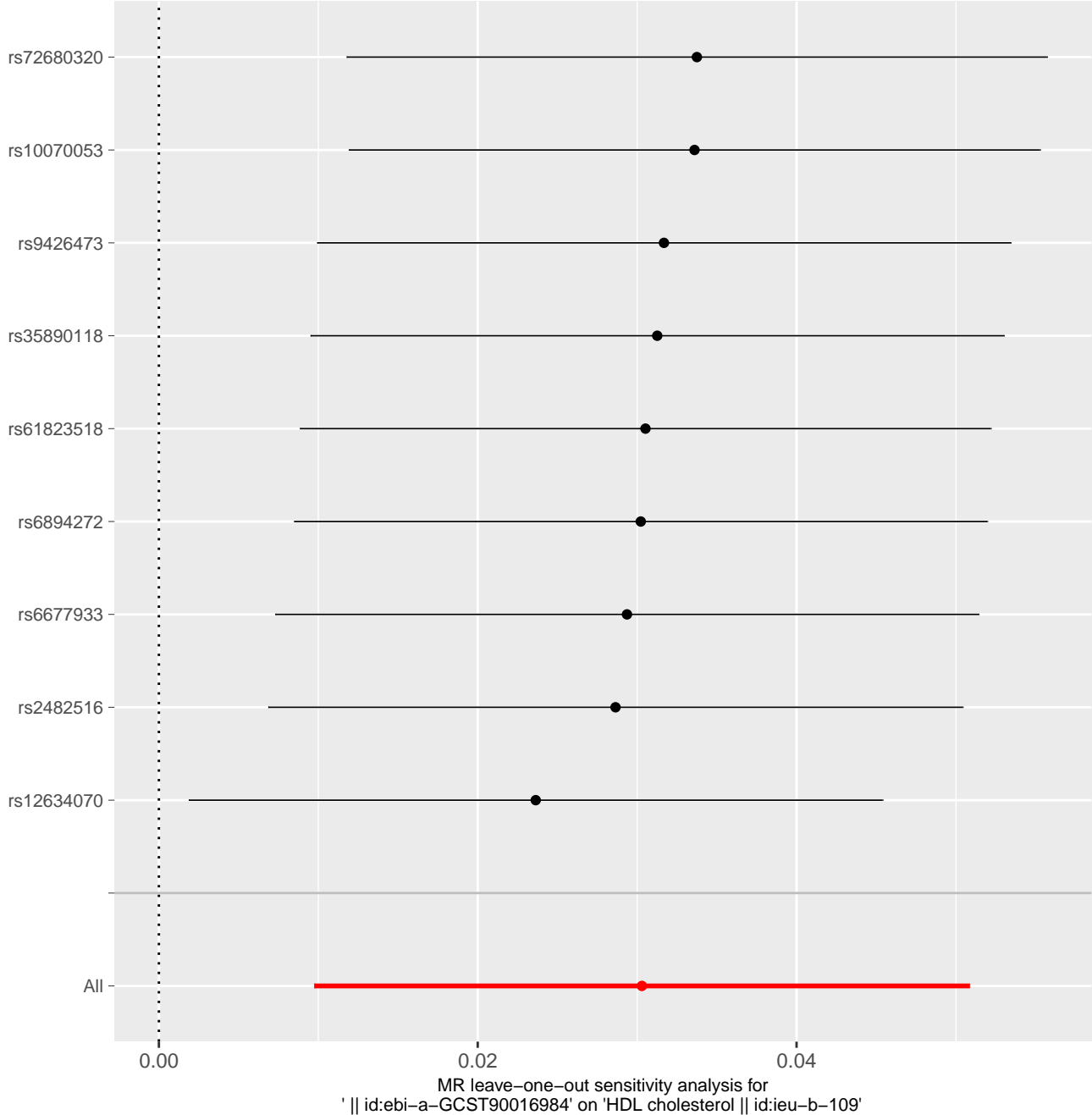

Supplement: Supplementary file 1 [file nutrients-15-04445-s001.zip › Supplementary materials 2/Leaveoneout plot for gut microbiota on HDL-C/Leaveoneout plot for ebi-a-GCST90016984 on HDL-C.pdf]

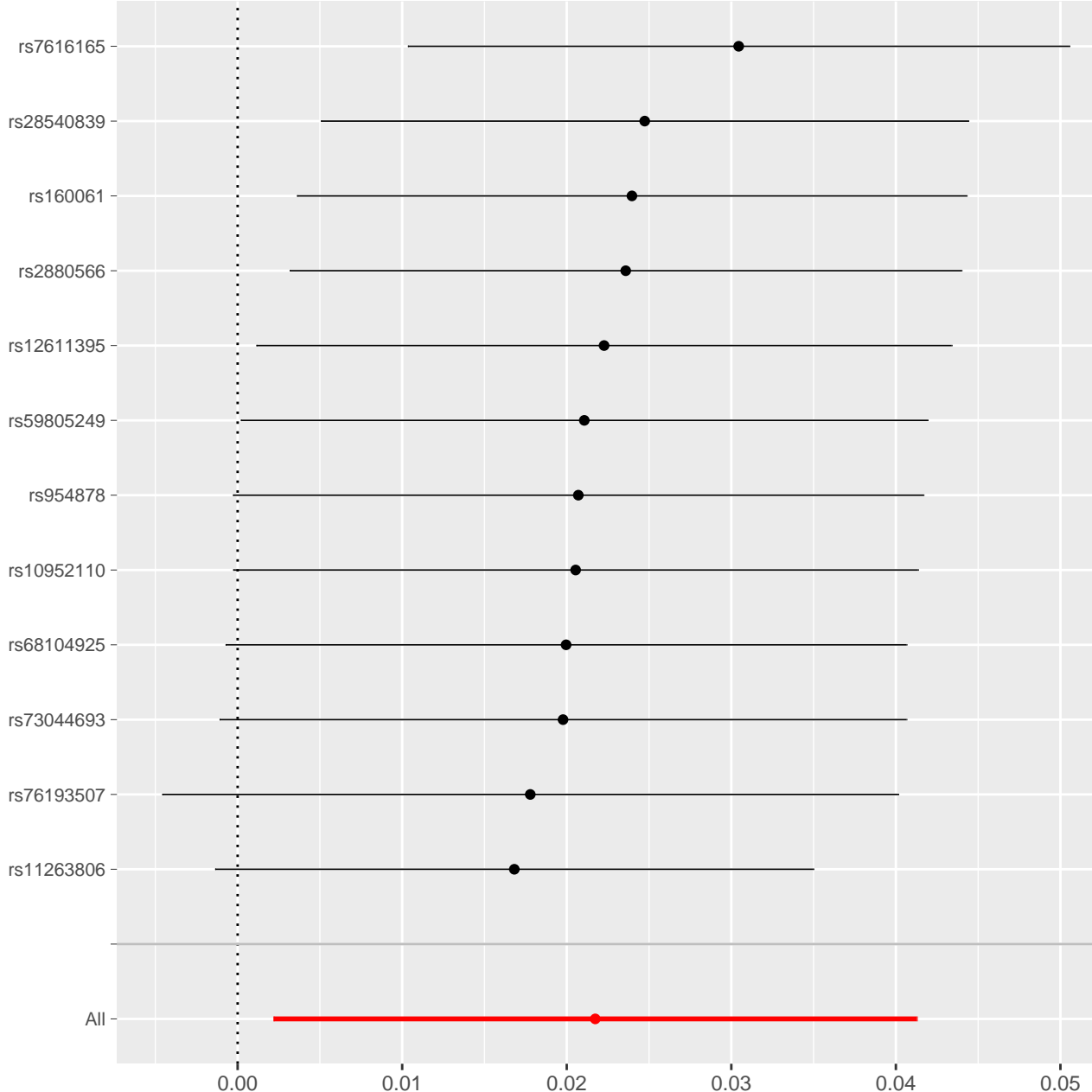

MR leave-one-out sensitivity analysis for  
' || id:ebi-a-GCST90017024' on 'HDL cholesterol || id:ieu-b-109'

Supplement: Supplementary file 1 [file nutrients-15-04445-s001.zip › Supplementary materials 2/Leaveoneout plot for gut microbiota on HDL-C/Leaveoneout plot for ebi-a-GCST90017024 on HDL-C.pdf]

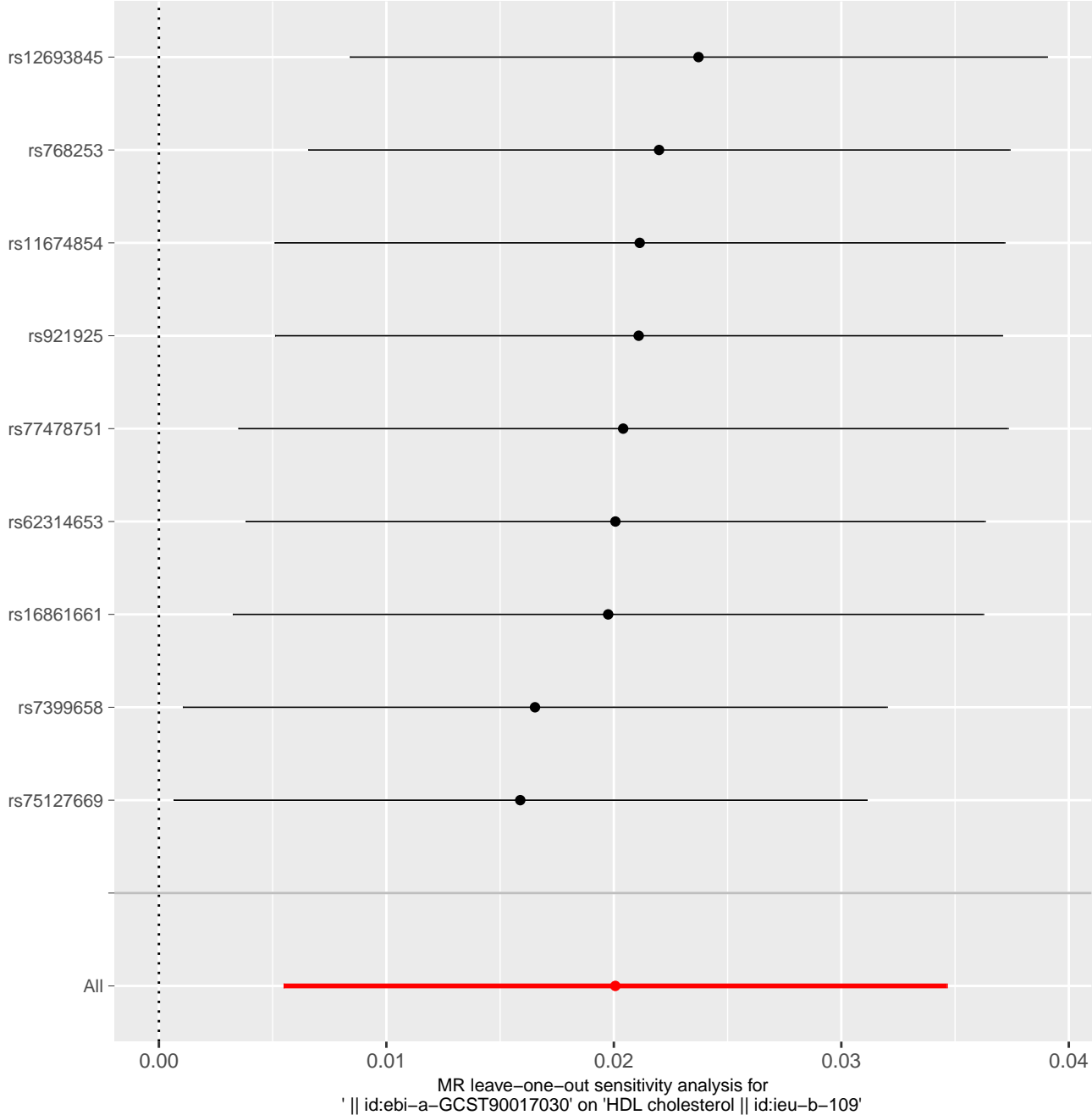

Supplement: Supplementary file 1 [file nutrients-15-04445-s001.zip › Supplementary materials 2/Leaveoneout plot for gut microbiota on HDL-C/Leaveoneout plot for ebi-a-GCST90017030 on HDL-C.pdf]

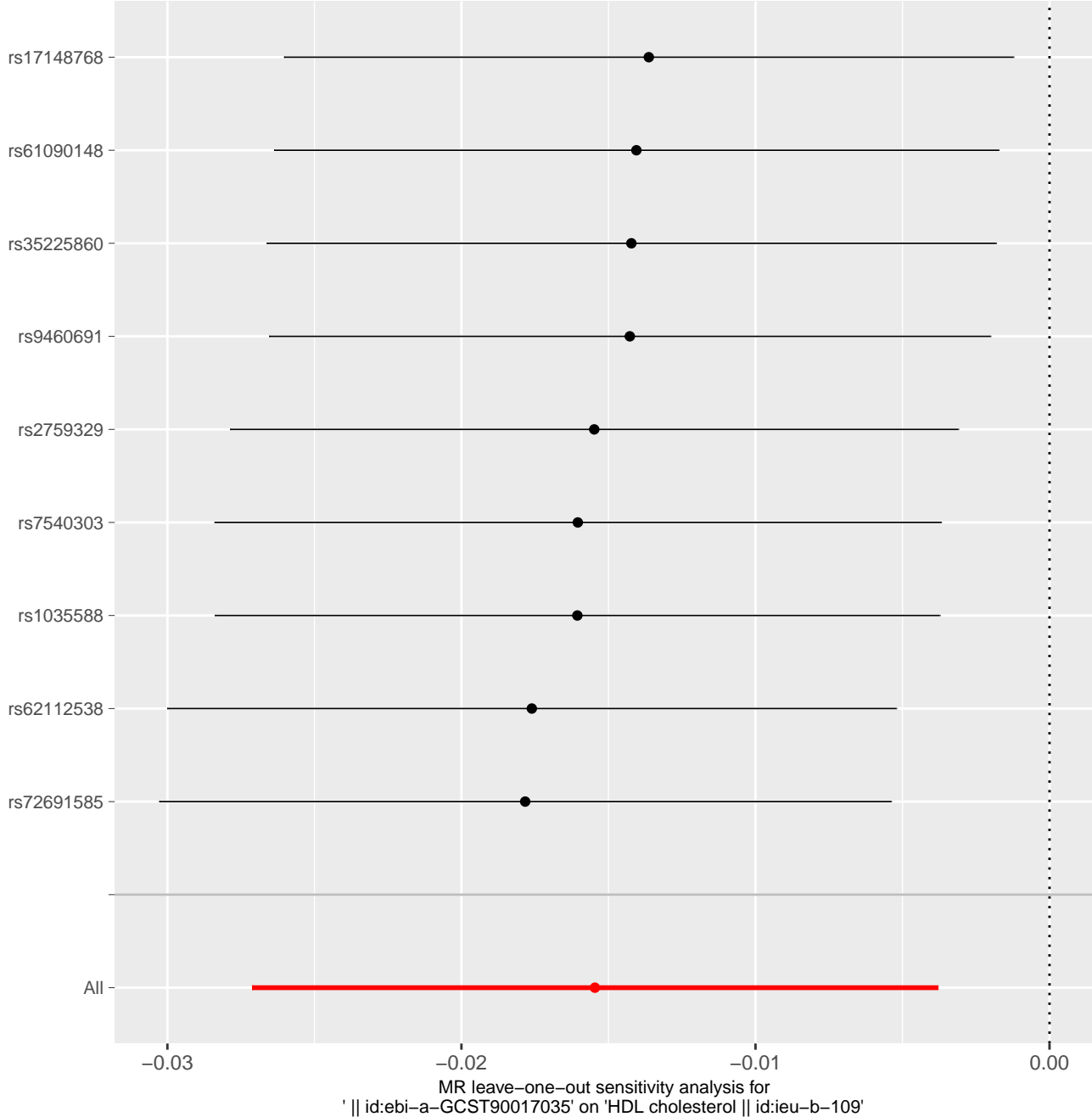

Supplement: Supplementary file 1 [file nutrients-15-04445-s001.zip › Supplementary materials 2/Leaveoneout plot for gut microbiota on HDL-C/Leaveoneout plot for ebi-a-GCST90017035 on HDL-C.pdf]

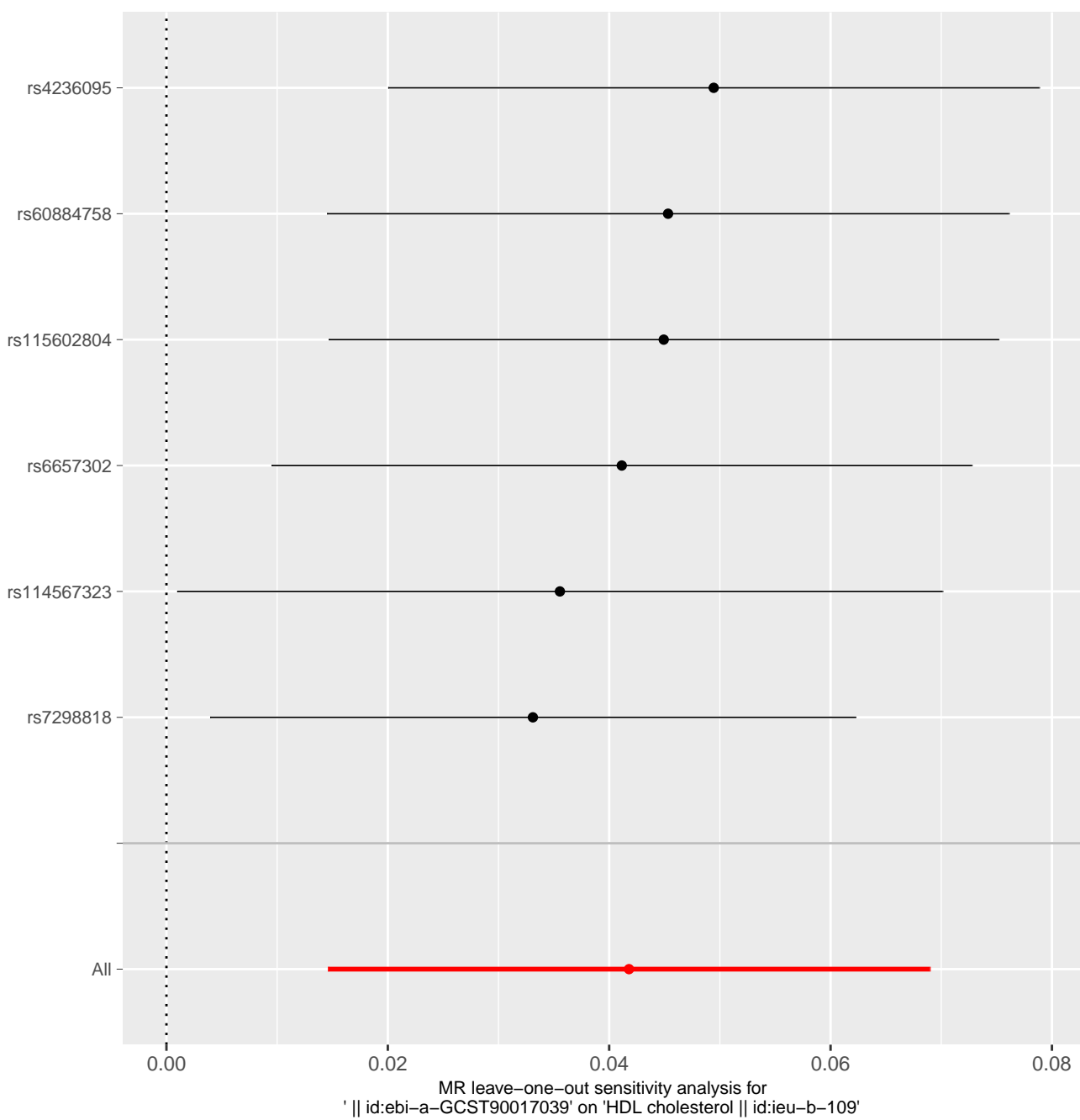

Supplement: Supplementary file 1 [file nutrients-15-04445-s001.zip › Supplementary materials 2/Leaveoneout plot for gut microbiota on HDL-C/Leaveoneout plot for ebi-a-GCST90017039 on HDL-C.pdf]

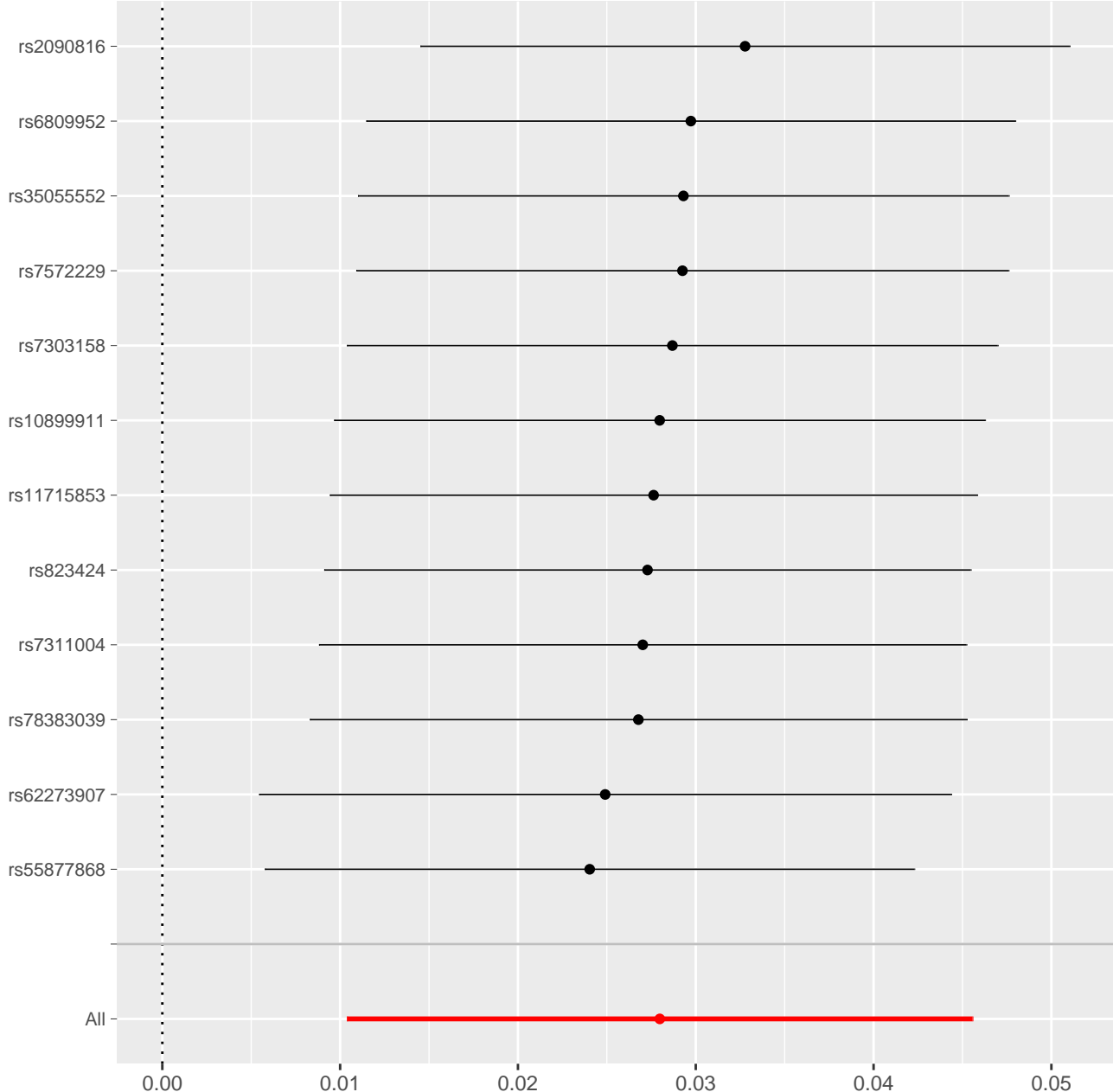

Supplement: Supplementary file 1 [file nutrients-15-04445-s001.zip › Supplementary materials 2/Leaveoneout plot for gut microbiota on LDL-C/Leaveoneout plot for ebi-a-GCST90017041 on LDL-C.pdf]

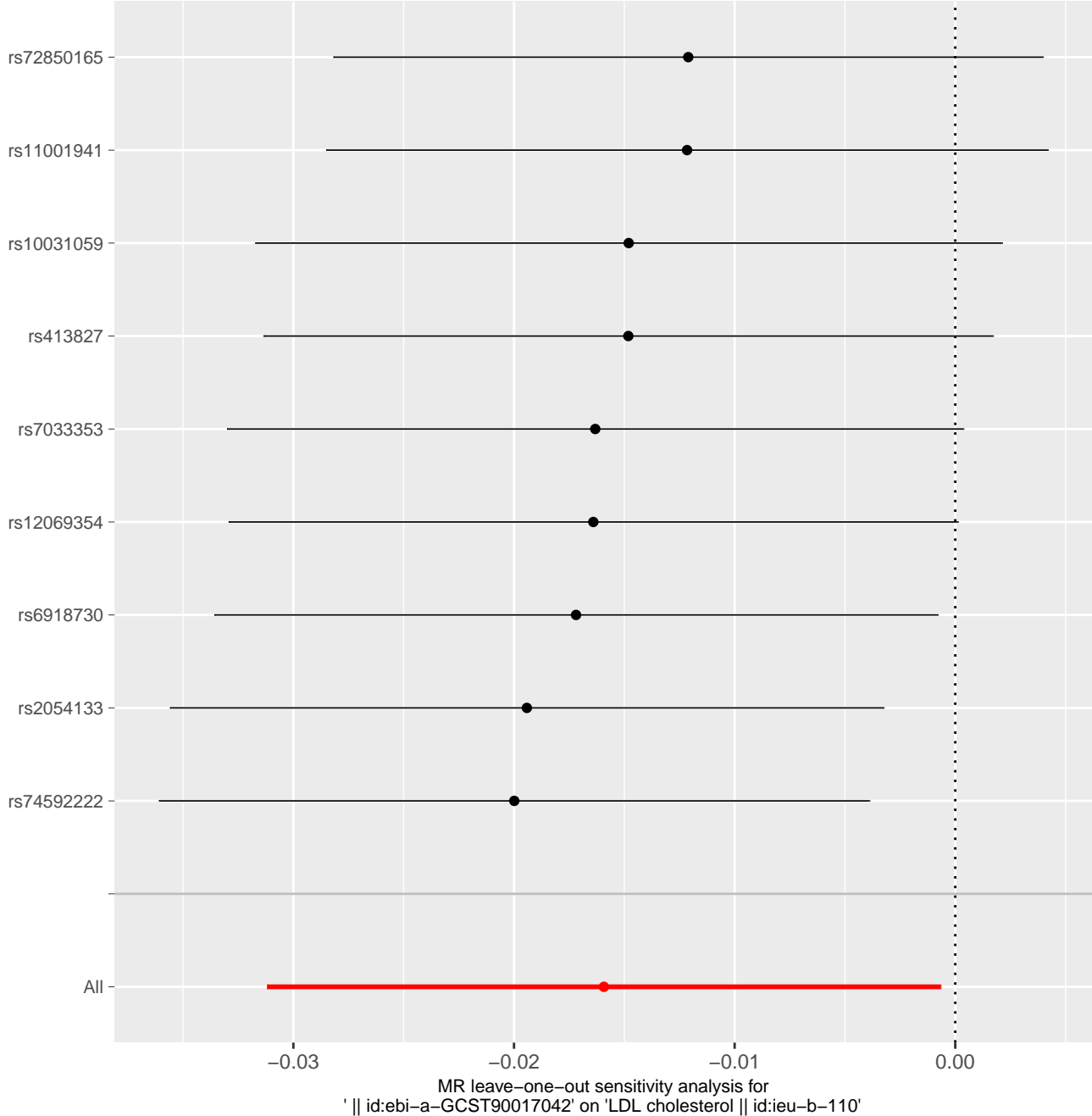

Supplement: Supplementary file 1 [file nutrients-15-04445-s001.zip › Supplementary materials 2/Leaveoneout plot for gut microbiota on LDL-C/Leaveoneout plot for ebi-a-GCST90017042 on LDL-C.pdf]

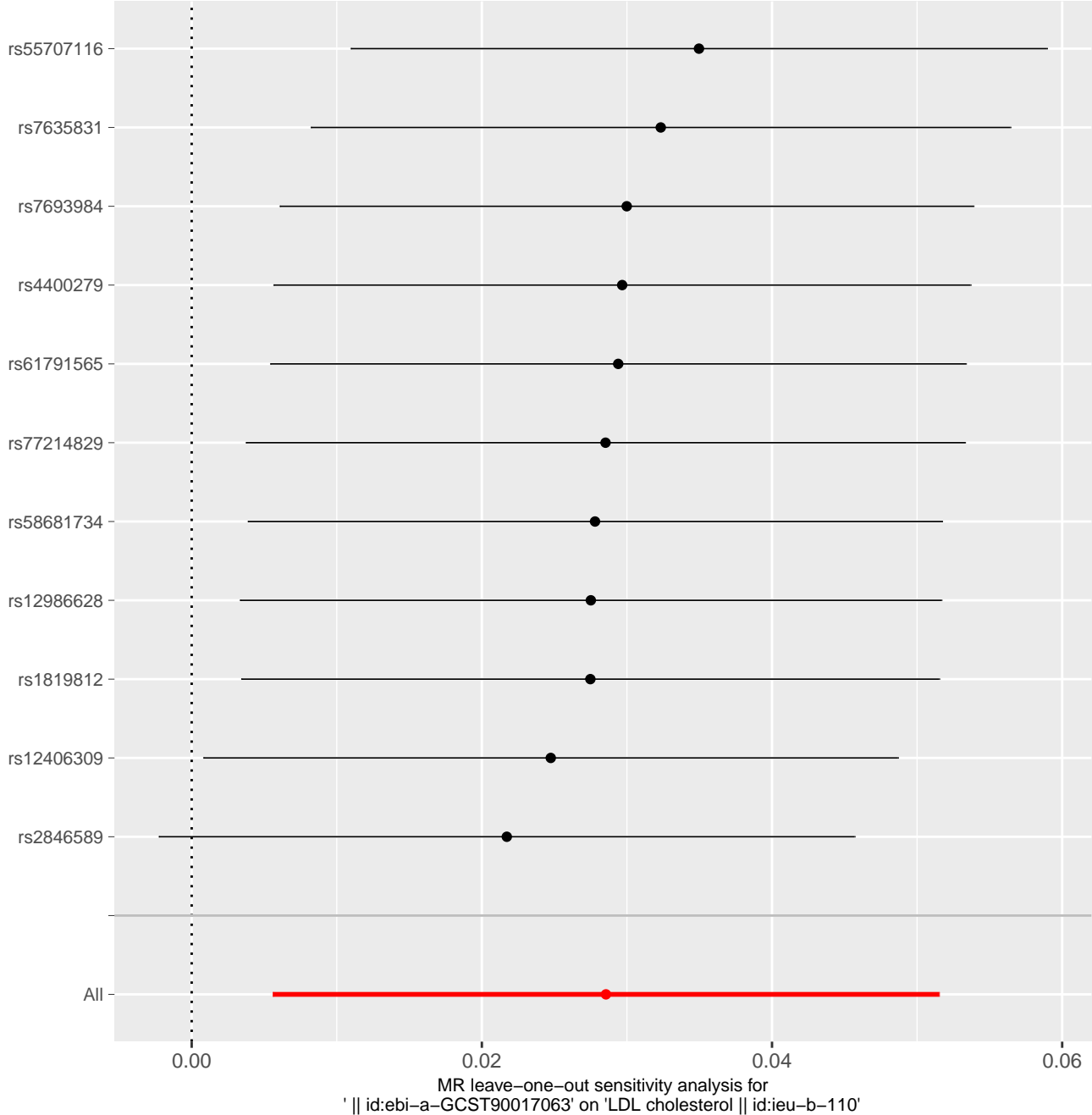

Supplement: Supplementary file 1 [file nutrients-15-04445-s001.zip › Supplementary materials 2/Leaveoneout plot for gut microbiota on LDL-C/Leaveoneout plot for ebi-a-GCST90017063 on LDL-C.pdf]

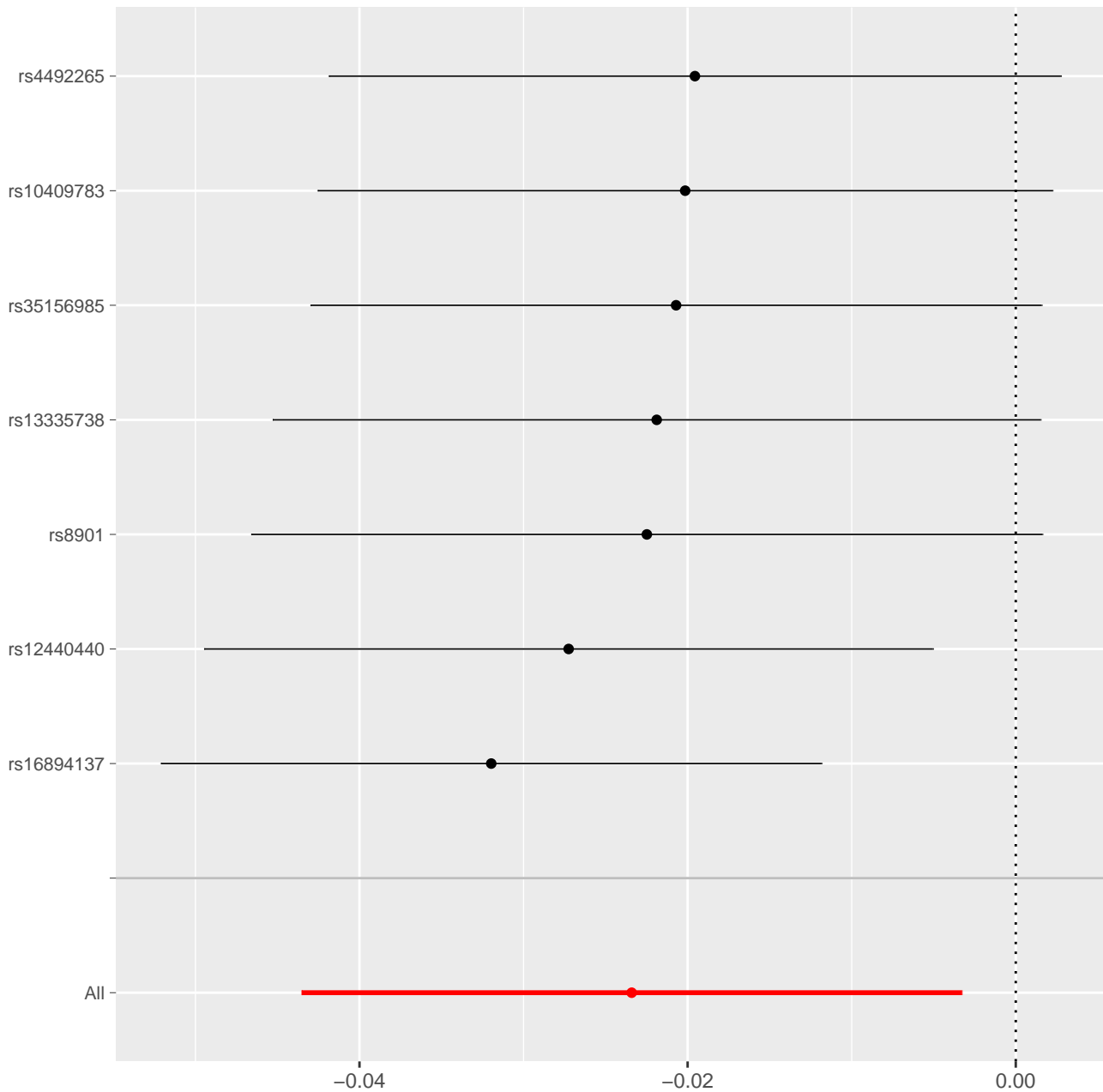

MR leave-one-out sensitivity analysis for  
' || id:ebi-a-GCST90017069' on 'LDL cholesterol || id:ieu-b-110'

Supplement: Supplementary file 1 [file nutrients-15-04445-s001.zip › Supplementary materials 2/Leaveoneout plot for gut microbiota on LDL-C/Leaveoneout plot for ebi-a-GCST90017069 on LDL-C.pdf]

rs58405430

rs1883097

rs2872237

rs7184125

All

0.000

0.025

0.050

0.075

MR leave-one-out sensitivity analysis for  
' || id:ebi-a-GCST90017073' on 'LDL cholesterol || id:ieu-b-110'

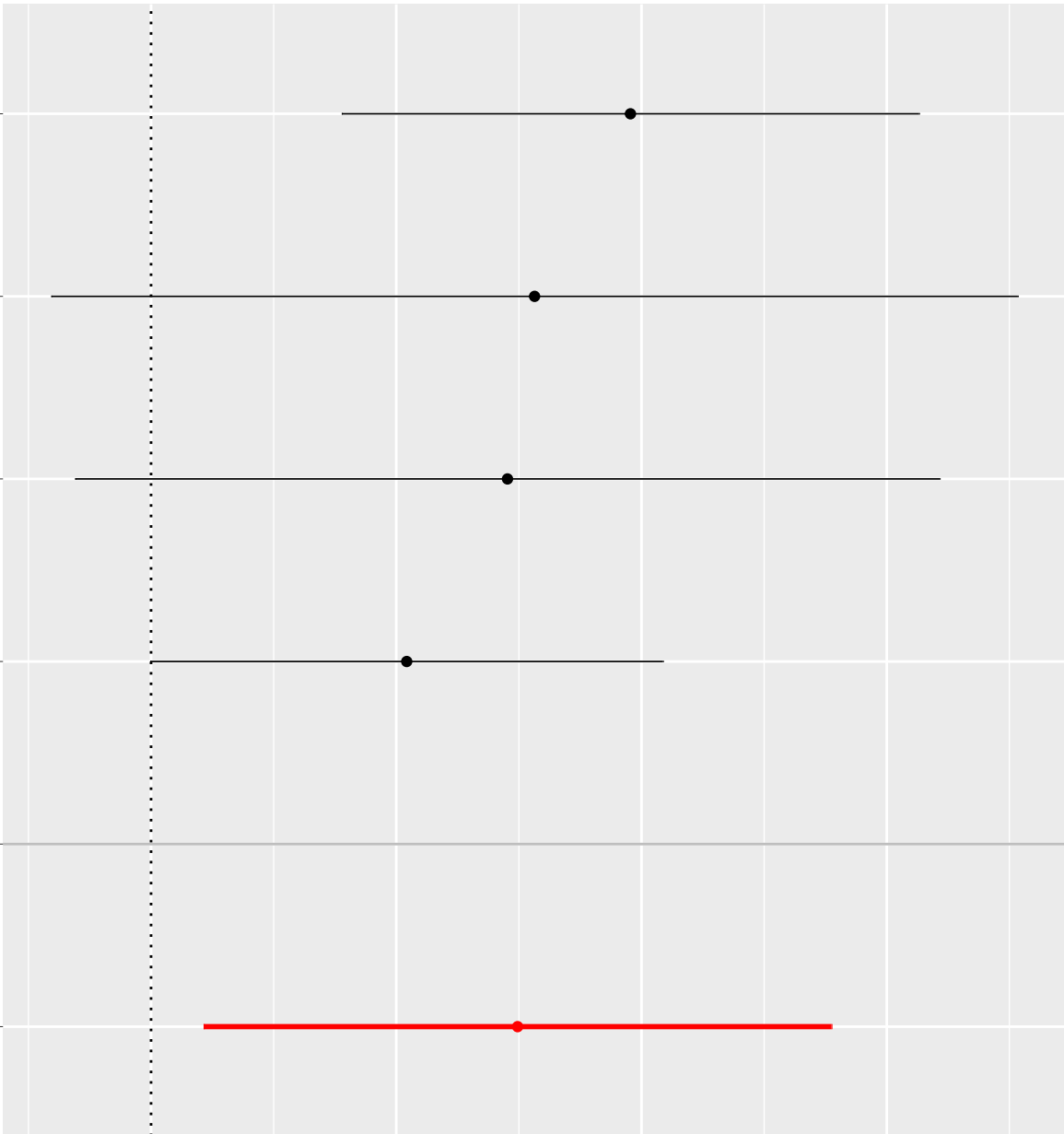

Supplement: Supplementary file 1 [file nutrients-15-04445-s001.zip › Supplementary materials 2/Leaveoneout plot for gut microbiota on LDL-C/Leaveoneout plot for ebi-a-GCST90017073 on LDL-C.pdf]

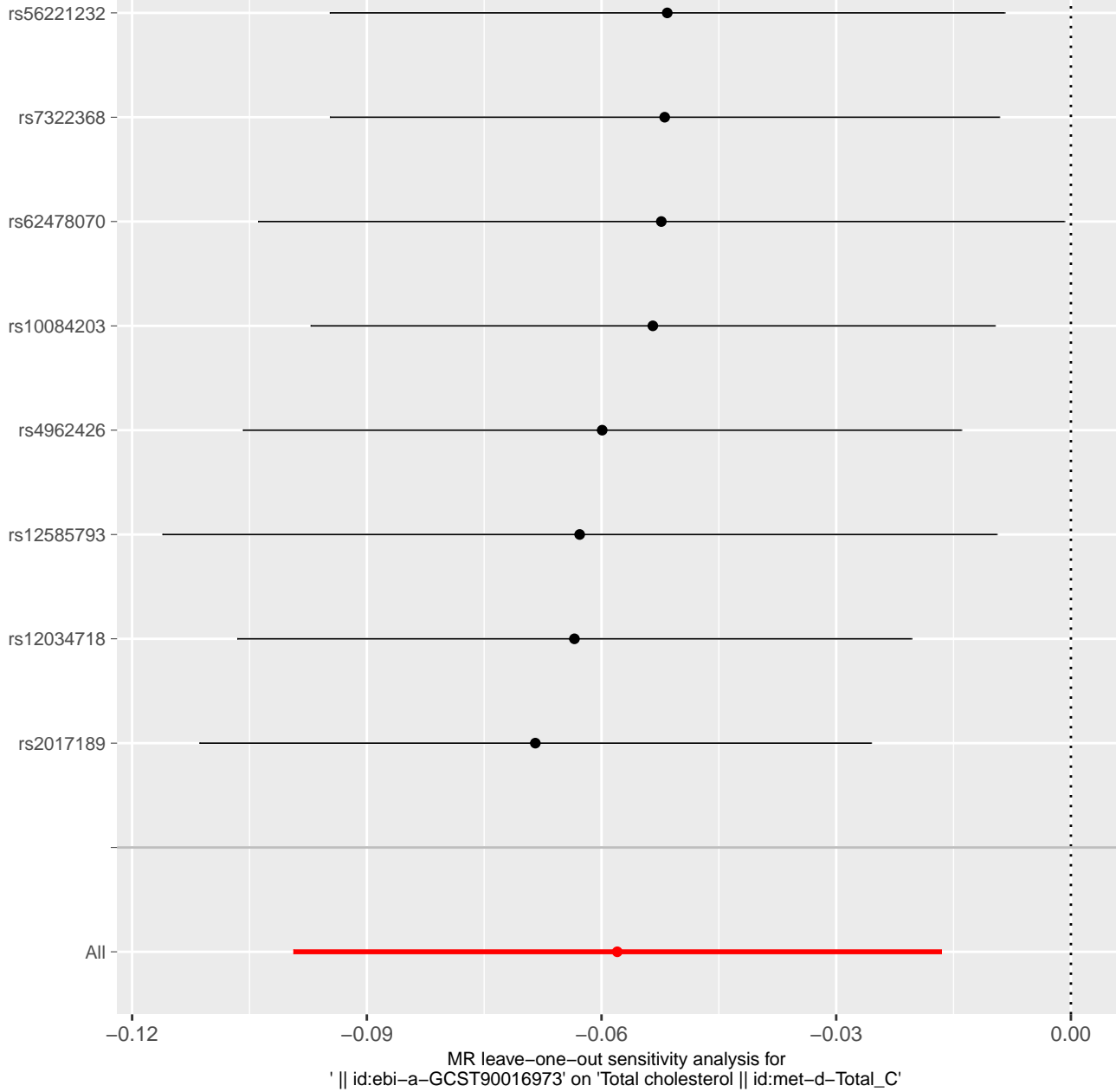

Supplement: Supplementary file 1 [file nutrients-15-04445-s001.zip › Supplementary materials 2/Leaveoneout plot for gut microbiota on TC/Leaveoneout plot for ebi-a-GCST90016973 on TC.pdf]

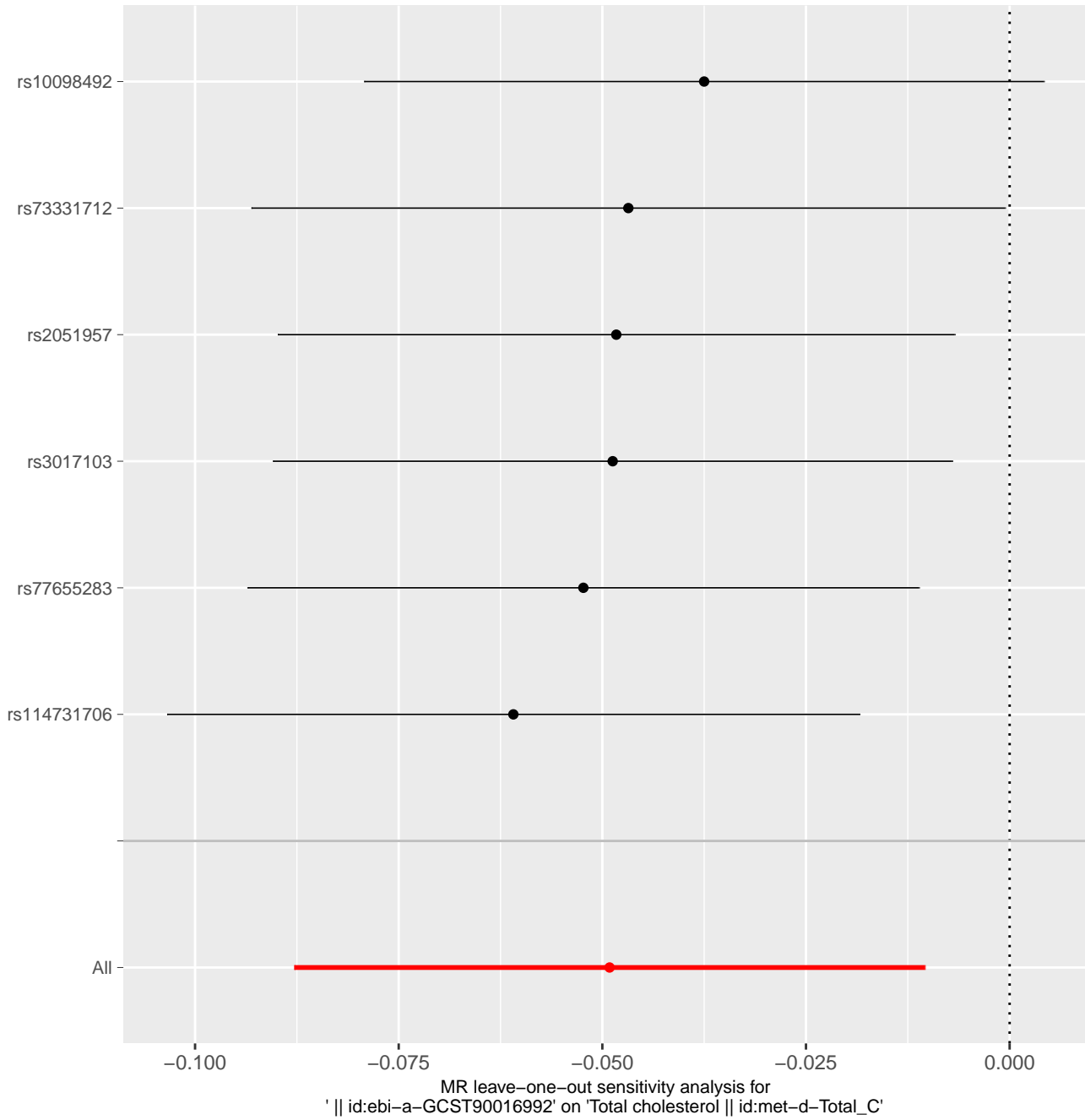

Supplement: Supplementary file 1 [file nutrients-15-04445-s001.zip › Supplementary materials 2/Leaveoneout plot for gut microbiota on TC/Leaveoneout plot for ebi-a-GCST90016992 on TC.pdf]

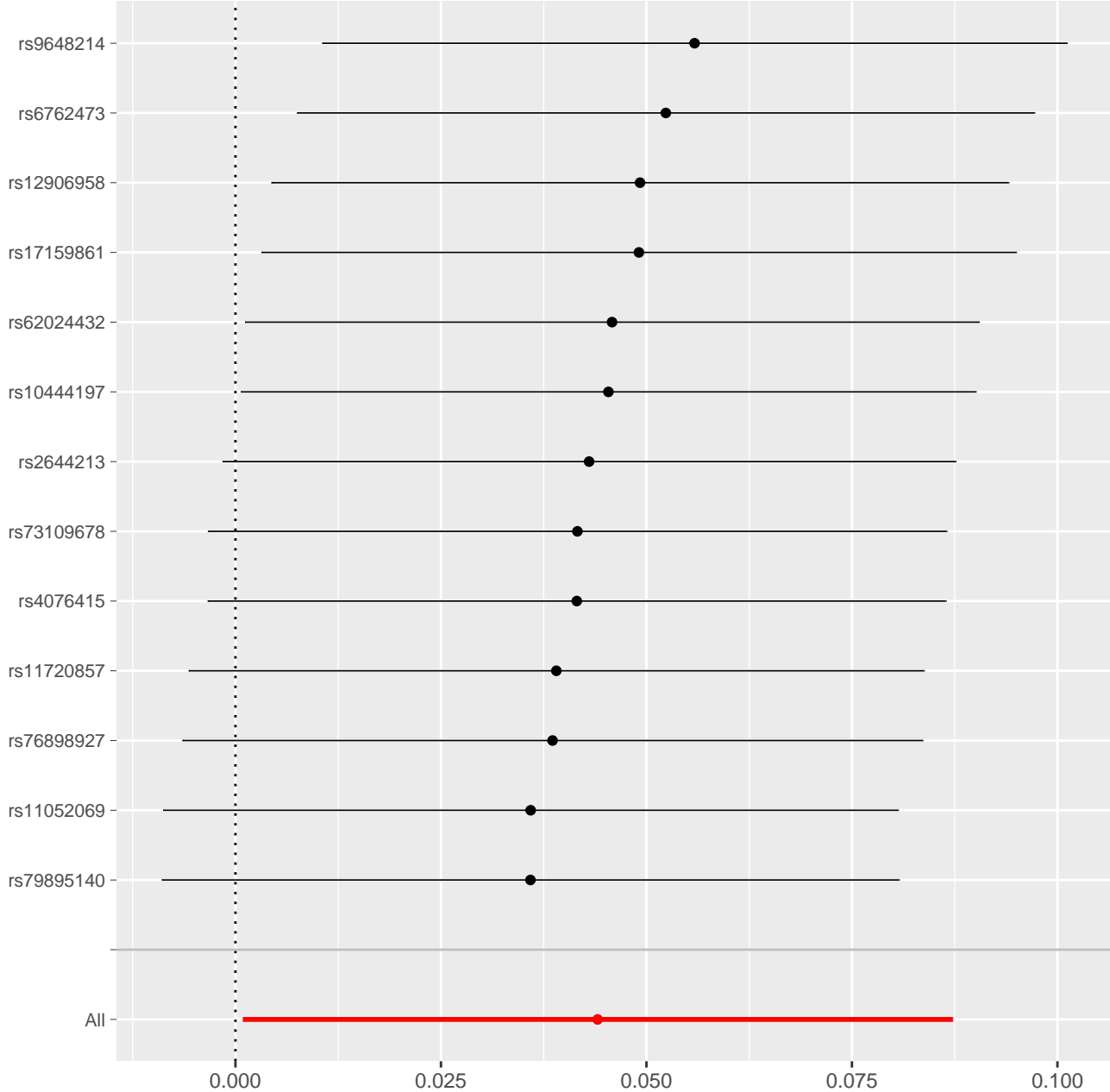

Supplement: Supplementary file 1 [file nutrients-15-04445-s001.zip › Supplementary materials 2/Leaveoneout plot for gut microbiota on TC/Leaveoneout plot for ebi-a-GCST90016997 on TC.pdf]

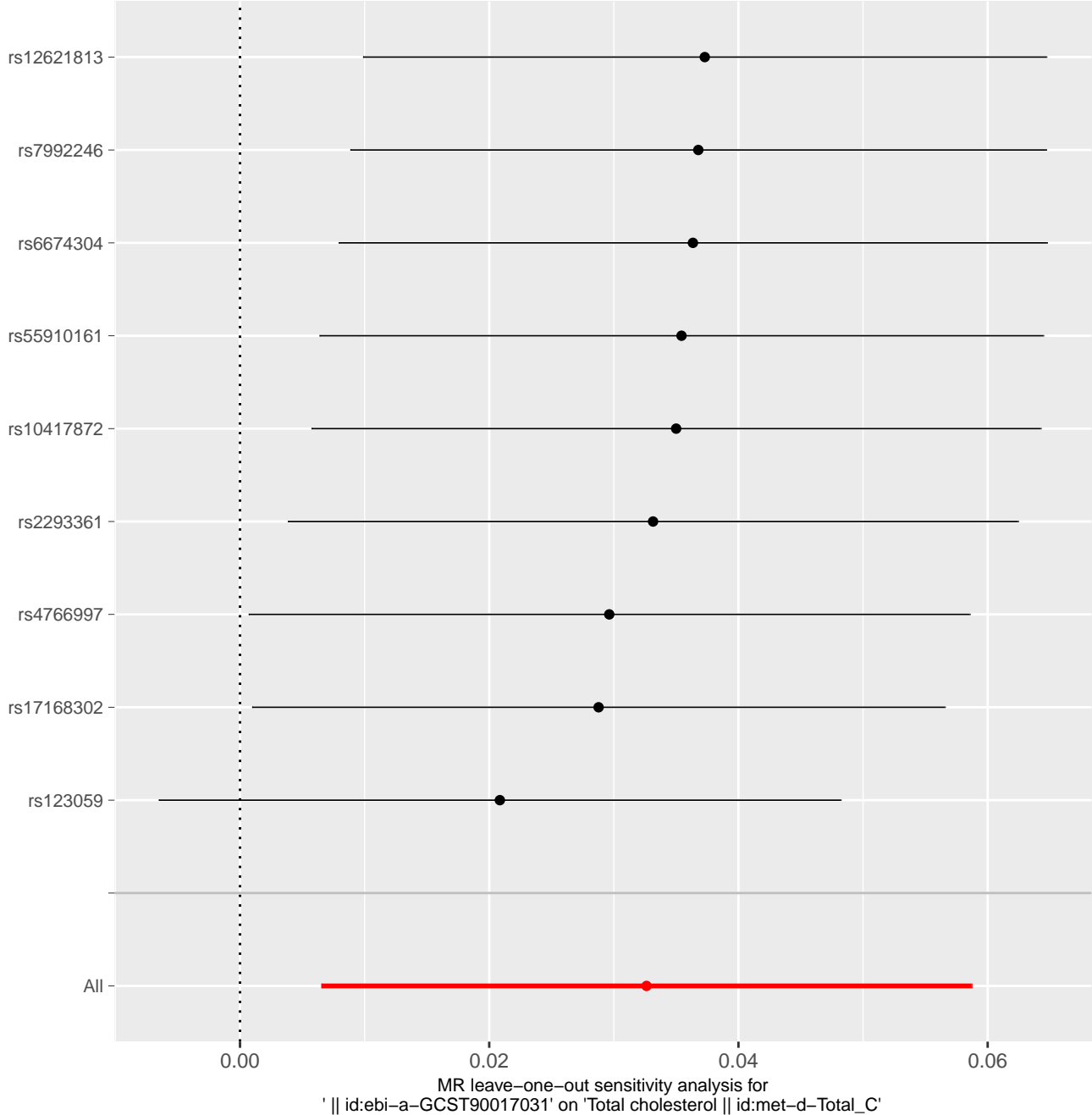

Supplement: Supplementary file 1 [file nutrients-15-04445-s001.zip › Supplementary materials 2/Leaveoneout plot for gut microbiota on TC/Leaveoneout plot for ebi-a-GCST90017031 on TC.pdf]

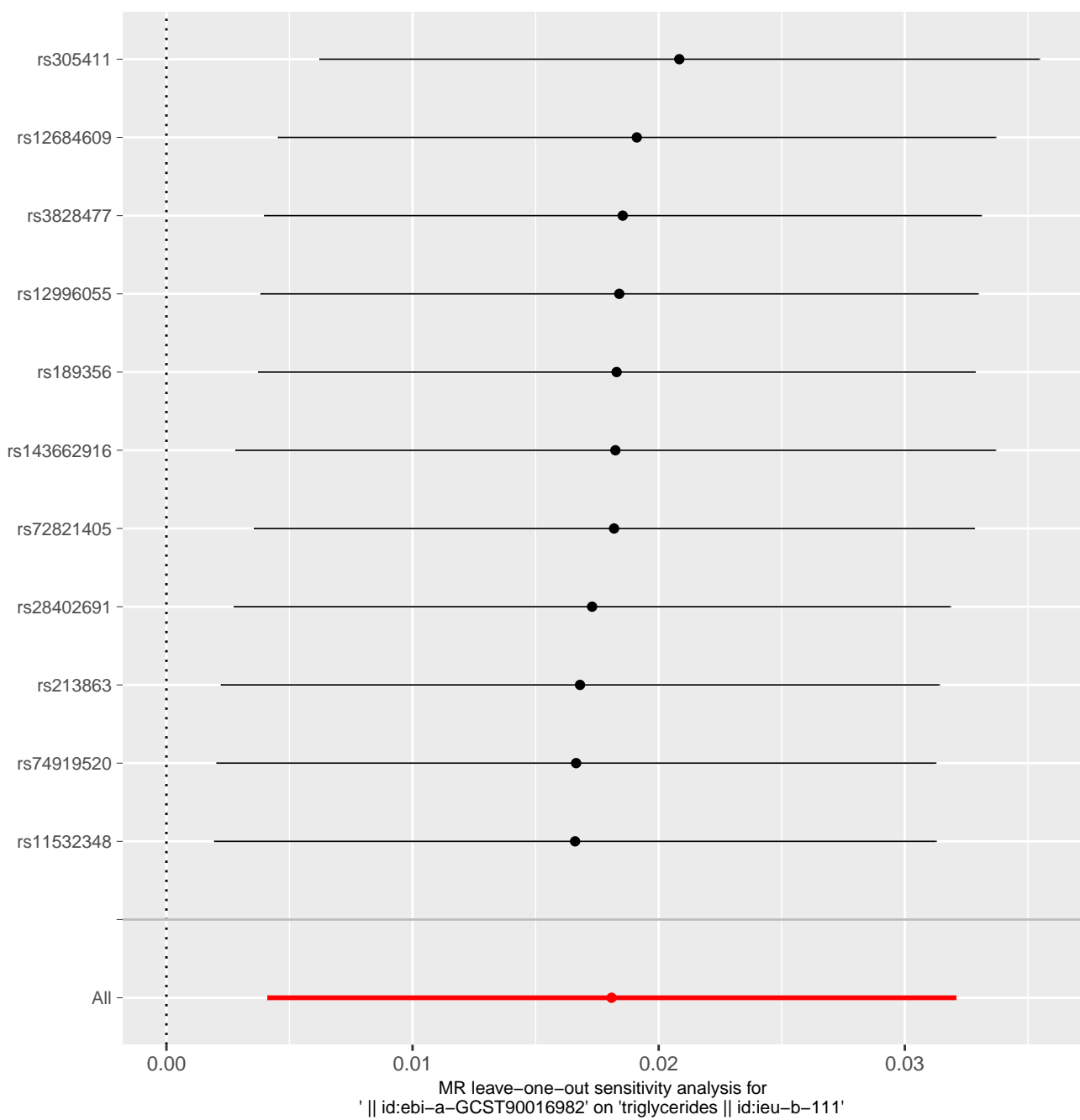

Supplement: Supplementary file 1 [file nutrients-15-04445-s001.zip › Supplementary materials 2/Leaveoneout plot for gut microbiota on TG/Leaveoneout plot for ebi-a-GCST90016982 on TG.pdf]

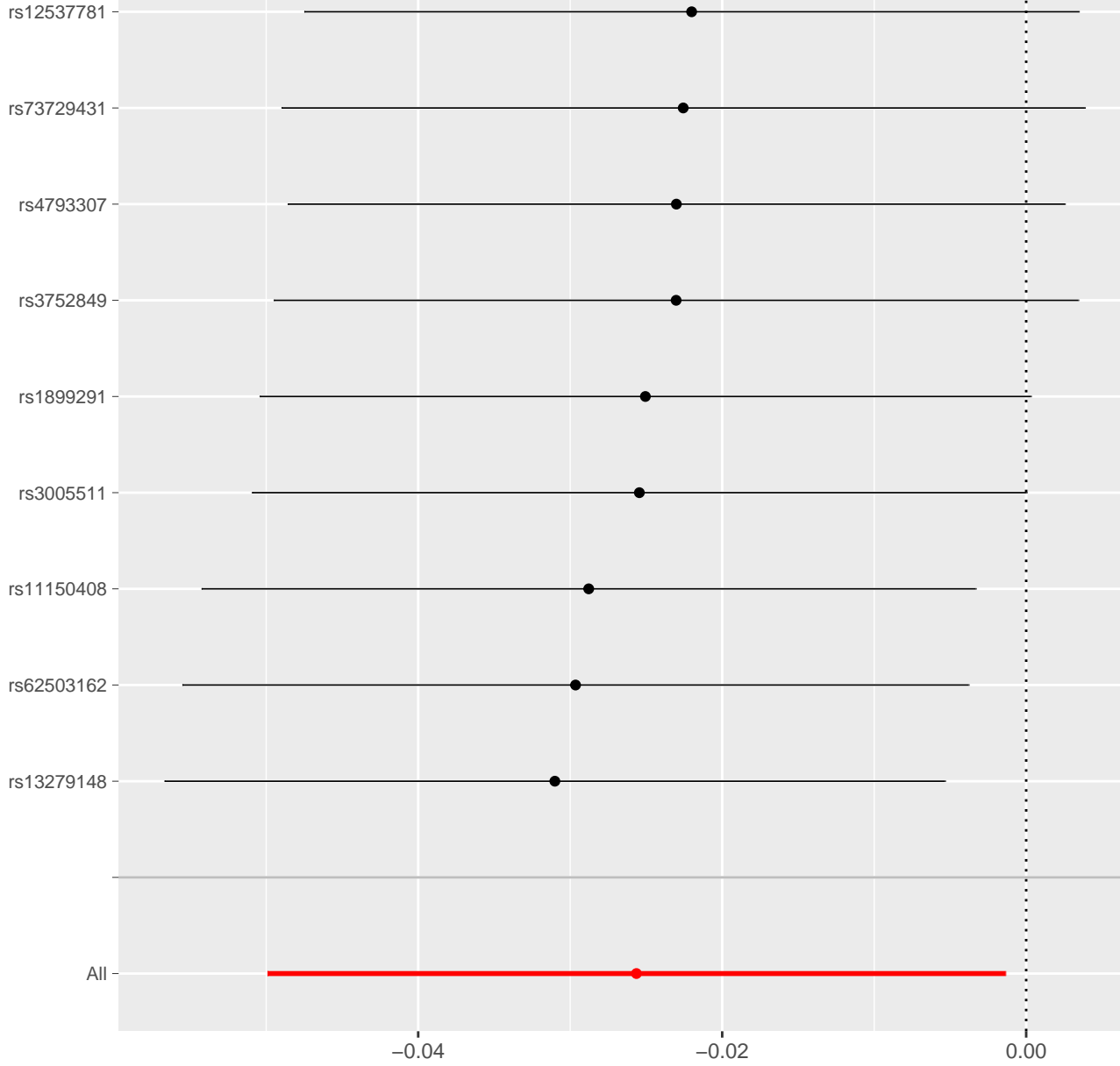

Supplement: Supplementary file 1 [file nutrients-15-04445-s001.zip › Supplementary materials 2/Leaveoneout plot for gut microbiota on TG/Leaveoneout plot for ebi-a-GCST90016989 on TG.pdf]

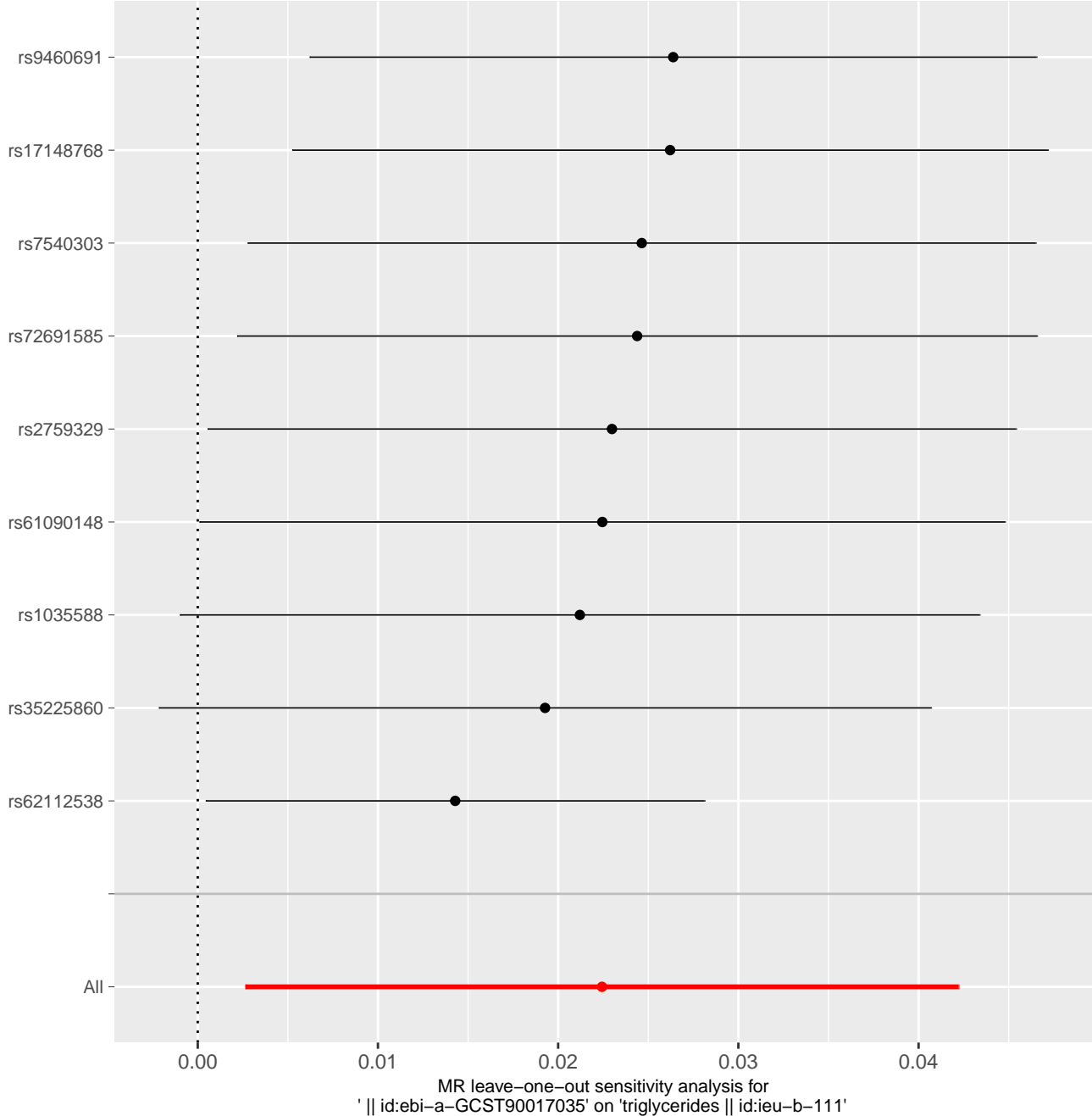

Supplement: Supplementary file 1 [file nutrients-15-04445-s001.zip › Supplementary materials 2/Leaveoneout plot for gut microbiota on TG/Leaveoneout plot for ebi-a-GCST90017035 on TG.pdf]

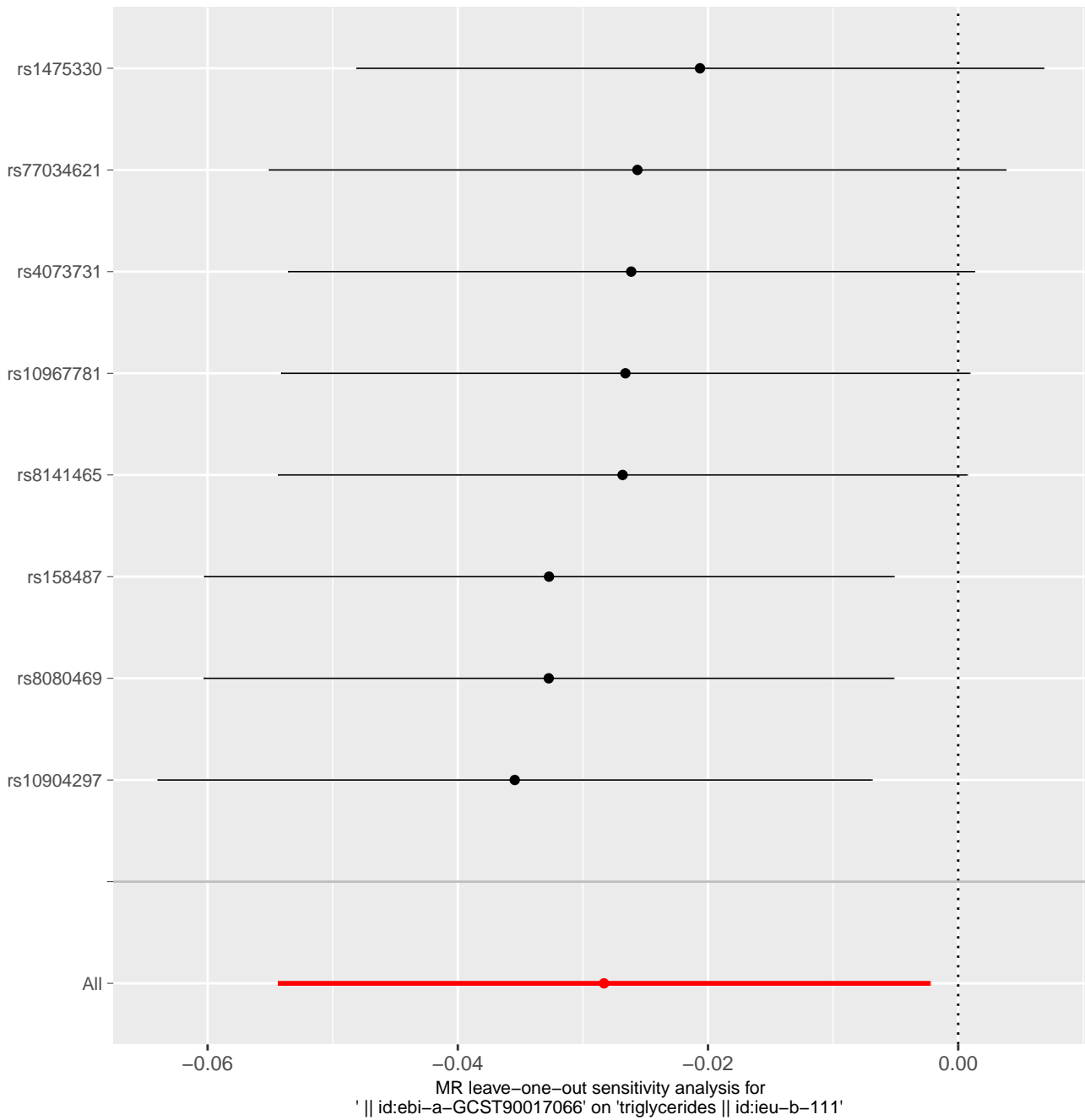

Supplement: Supplementary file 1 [file nutrients-15-04445-s001.zip › Supplementary materials 2/Leaveoneout plot for gut microbiota on TG/Leaveoneout plot for ebi-a-GCST90017066 on TG.pdf]

## Gut microbiota genera and HDL-C

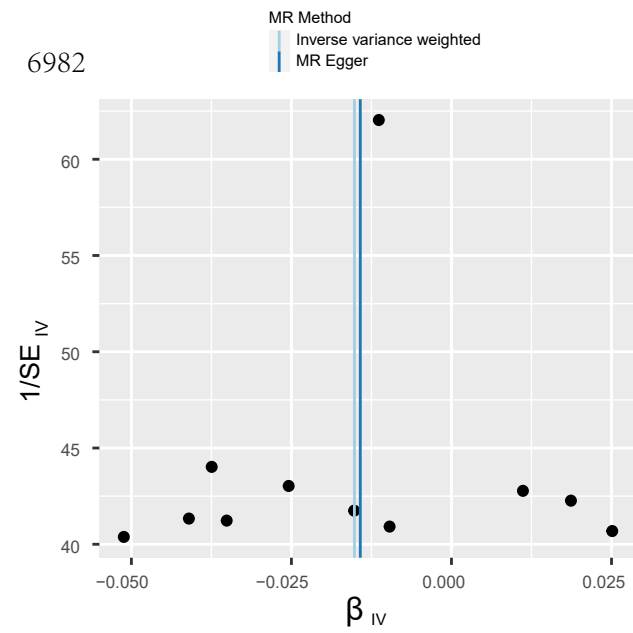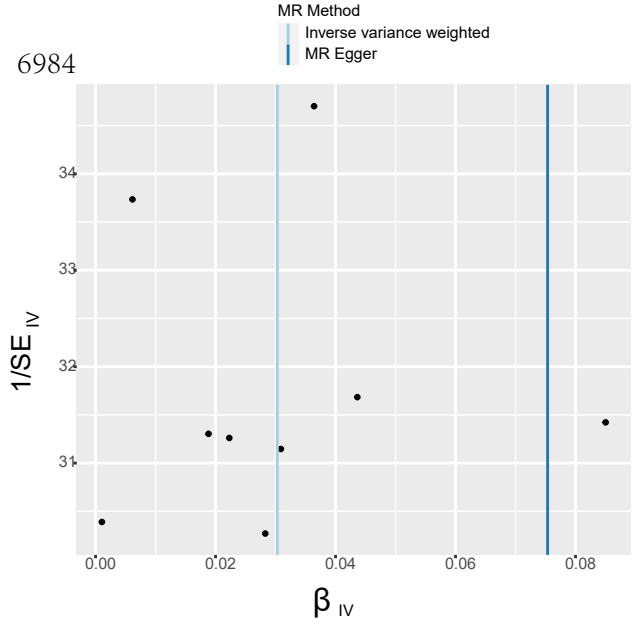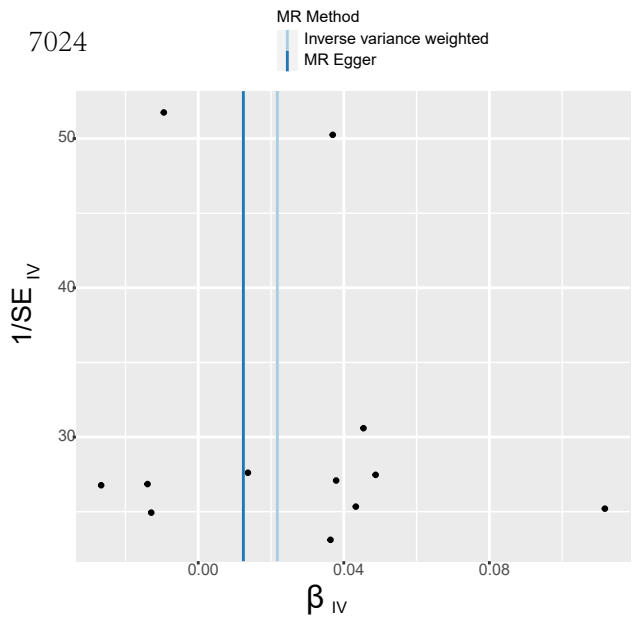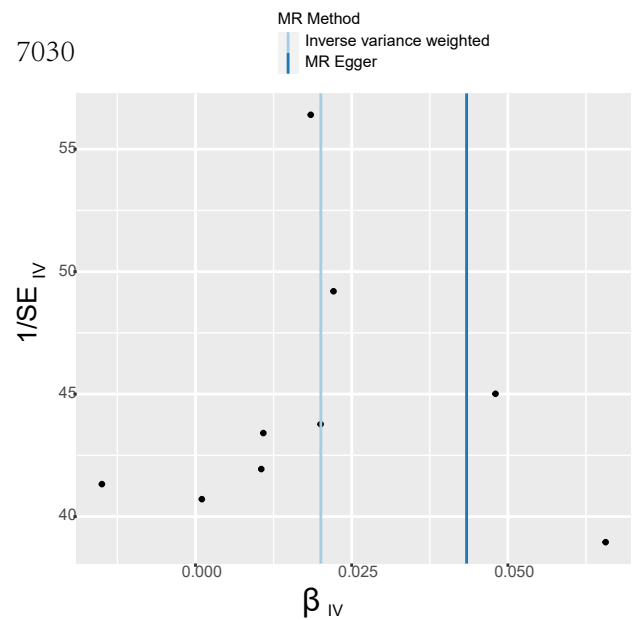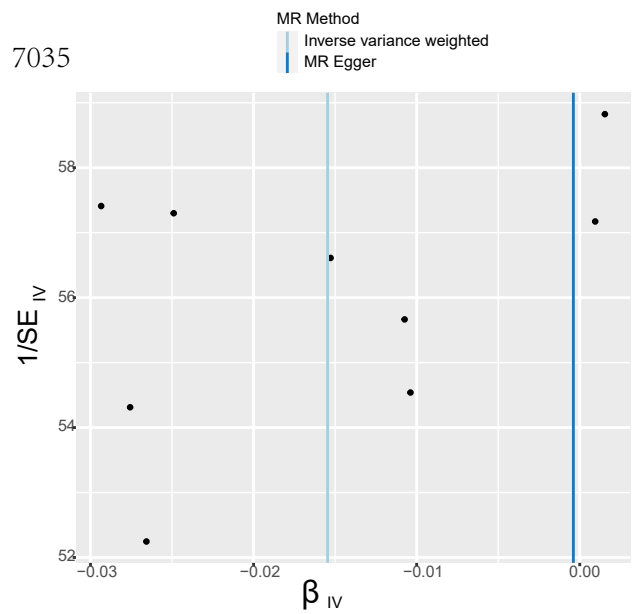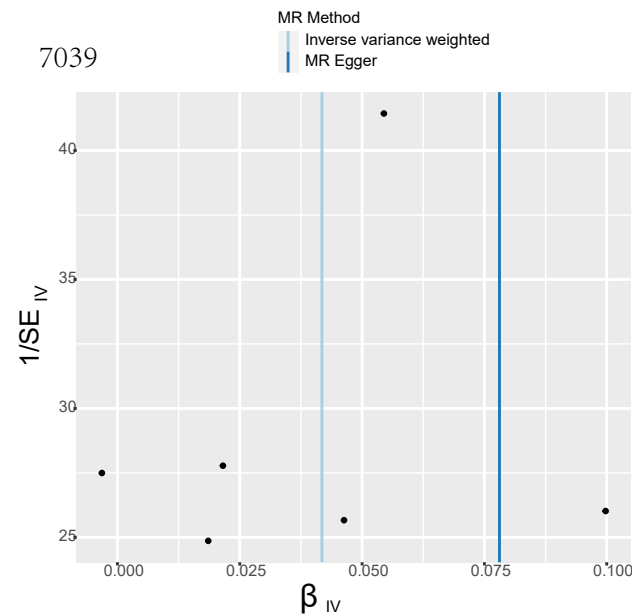

Supplement: Supplementary file 1 [file nutrients-15-04445-s001.zip › Supplementary materials 3/01.Funel plot for gut microbiota on HDL-C.pdf]

# Gut microbiota genera and LDL-C

7041

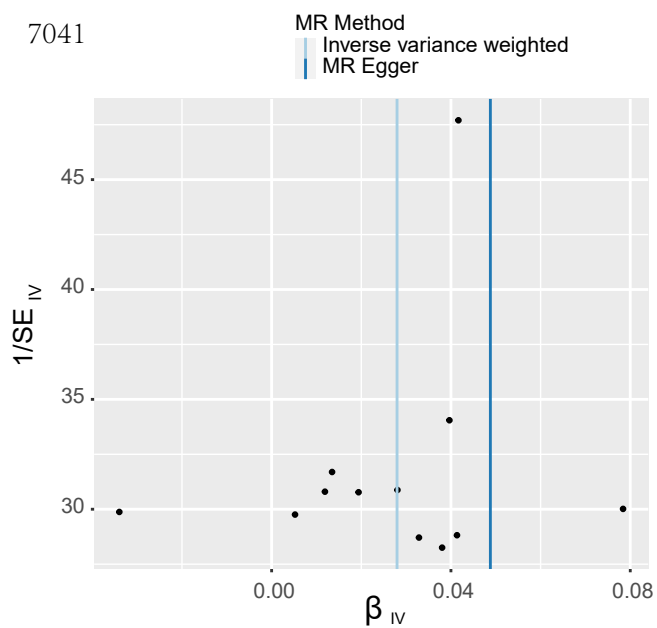

7042

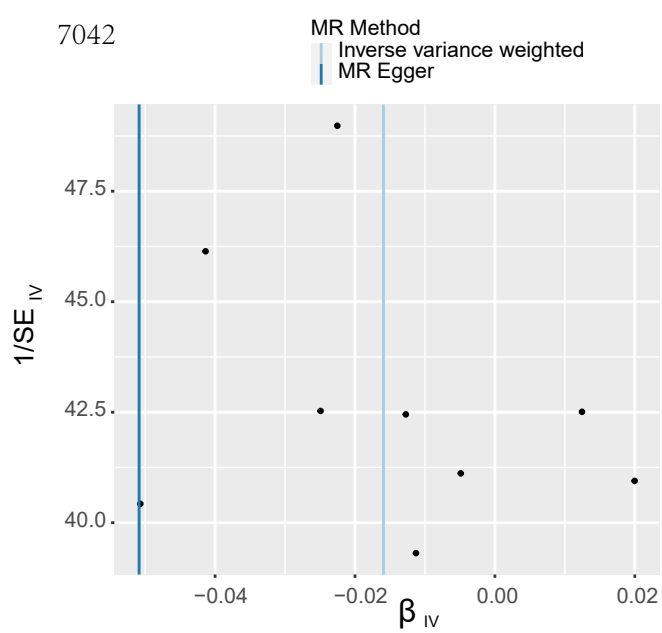

7063

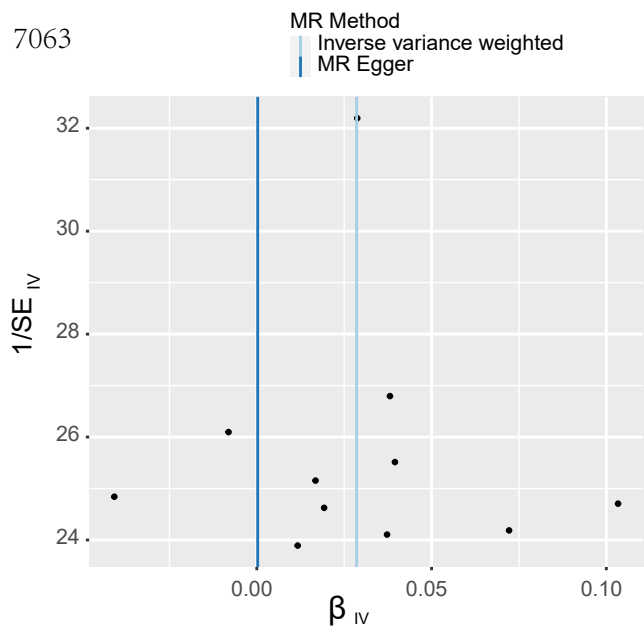

7069

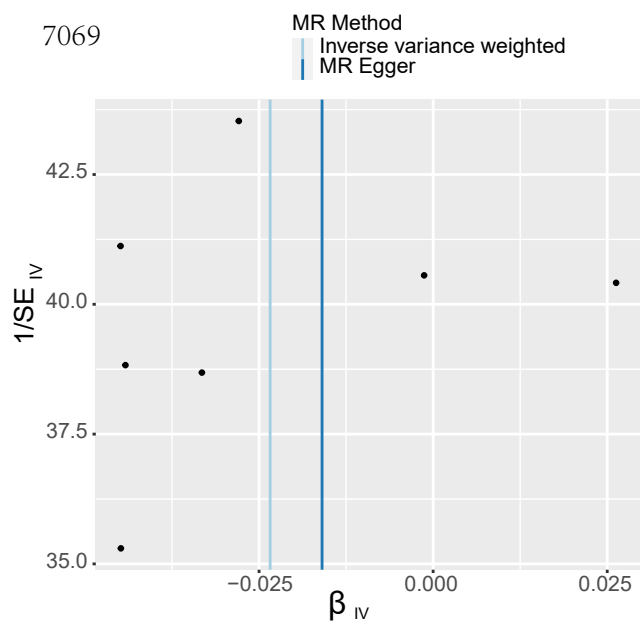

7073

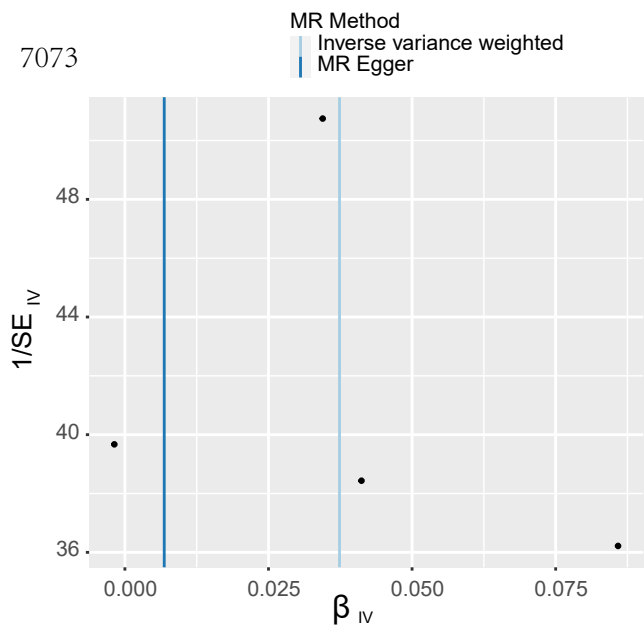

Supplement: Supplementary file 1 [file nutrients-15-04445-s001.zip › Supplementary materials 3/02.Funel plot for gut microbiota on LDL-C.pdf]

## Gut microbiota genera and TC

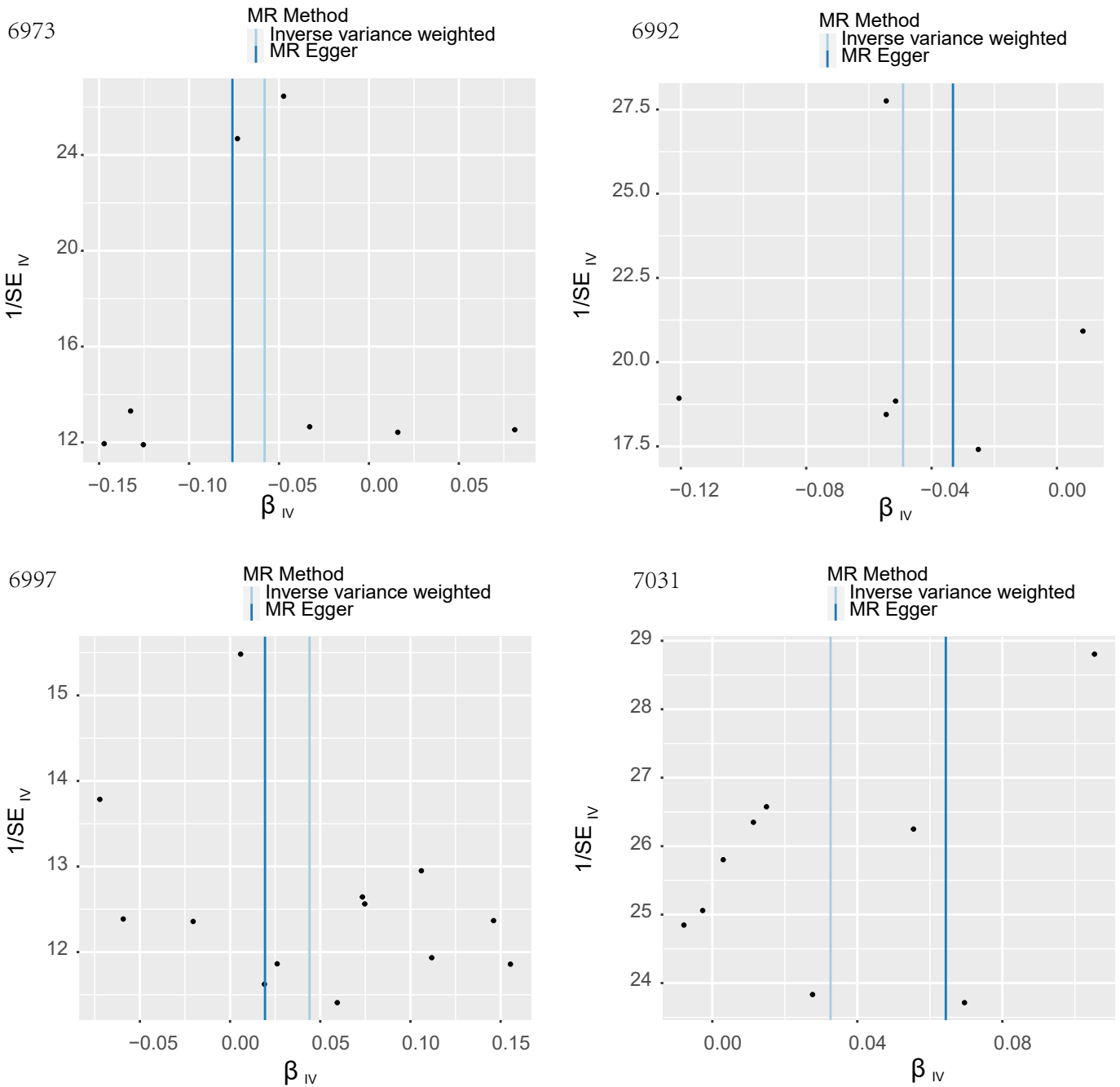

Supplement: Supplementary file 1 [file nutrients-15-04445-s001.zip › Supplementary materials 3/03.Funel plot for gut microbiota on TC.pdf]

## Gut microbiota genera and TG

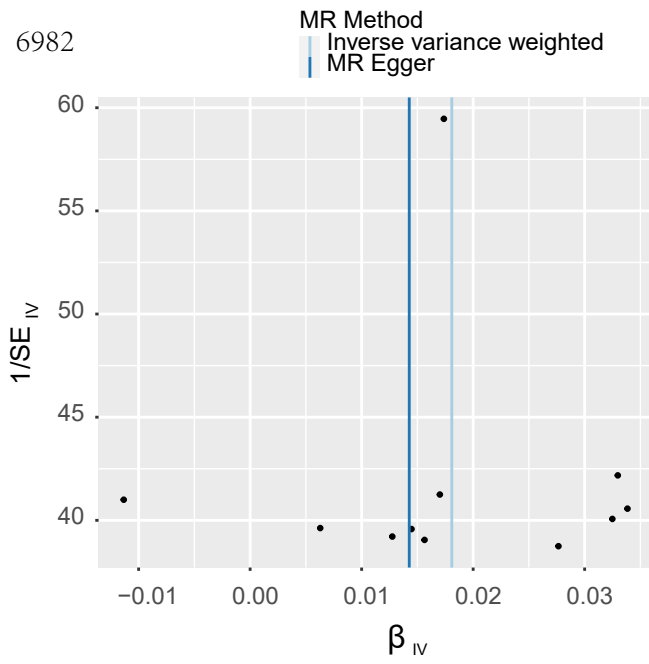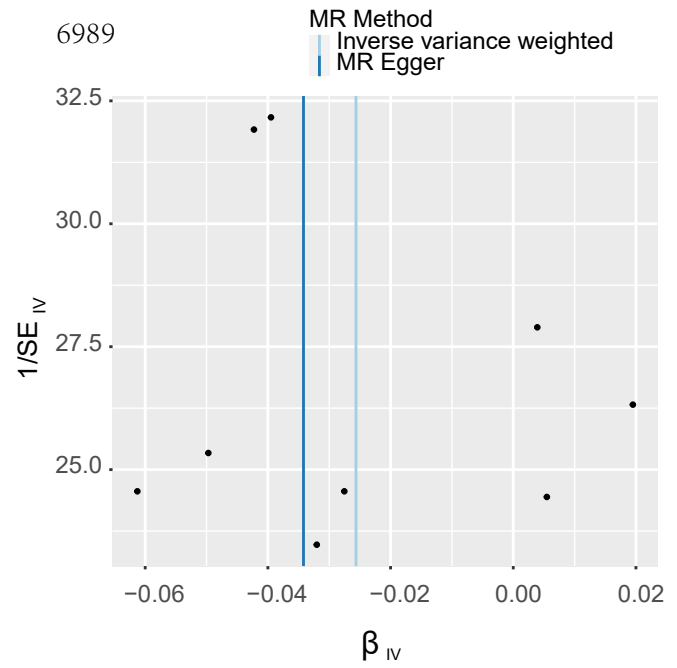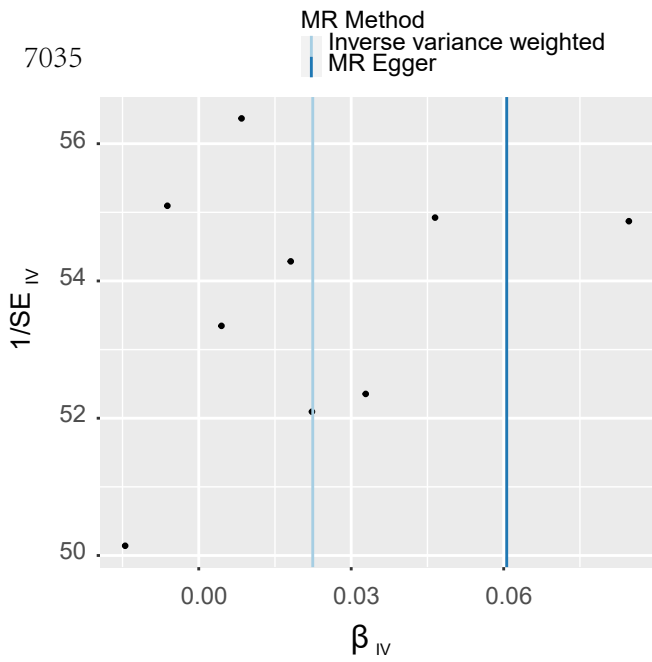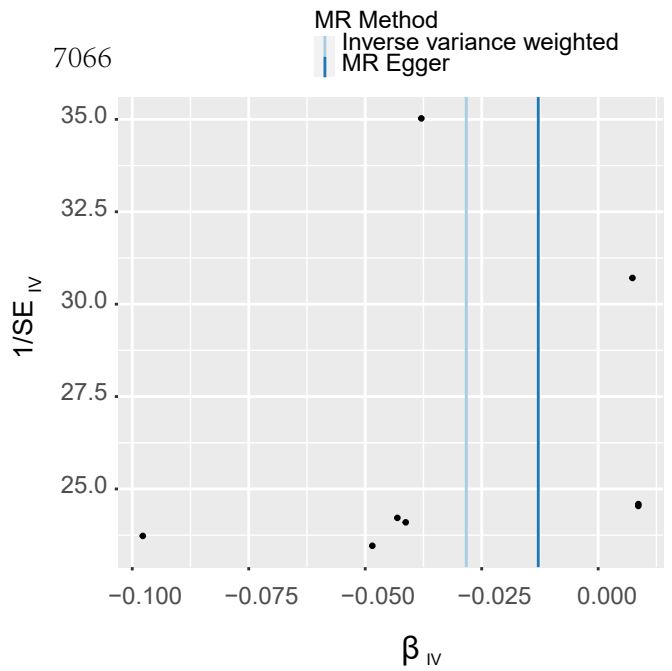

Supplement: Supplementary file 1 [file nutrients-15-04445-s001.zip › Supplementary materials 3/04.Funel plot for gut microbiota on TG.pdf]

Gut microbiota genera and APOA1

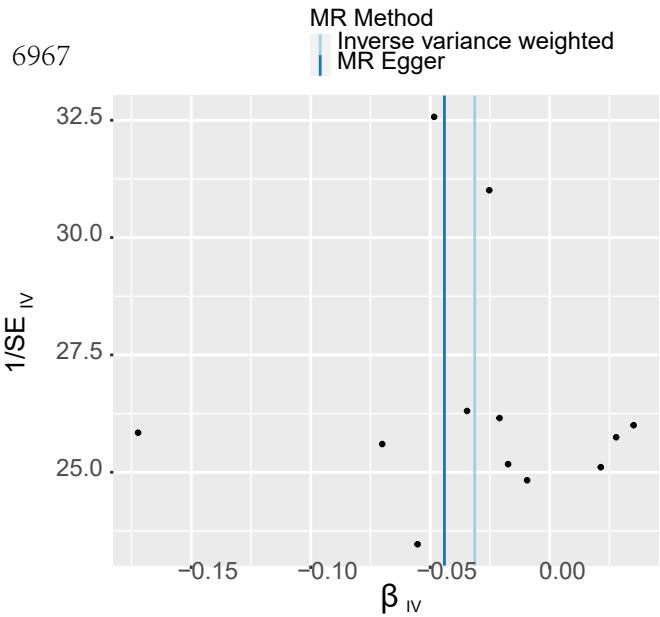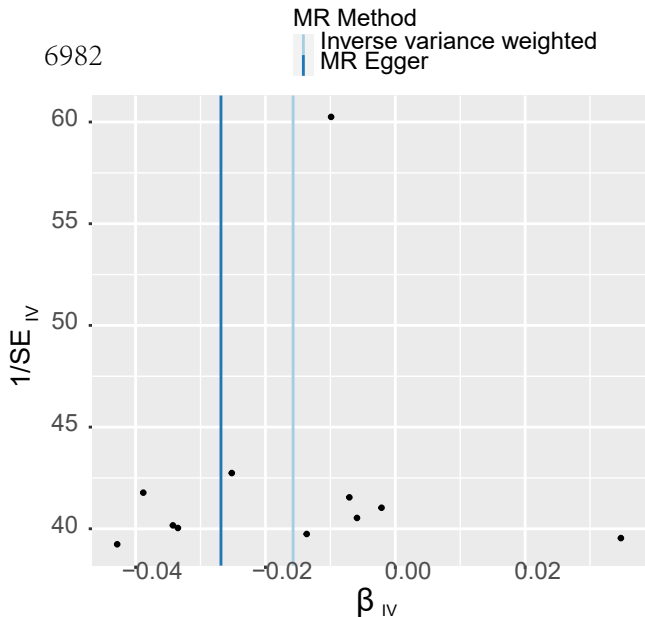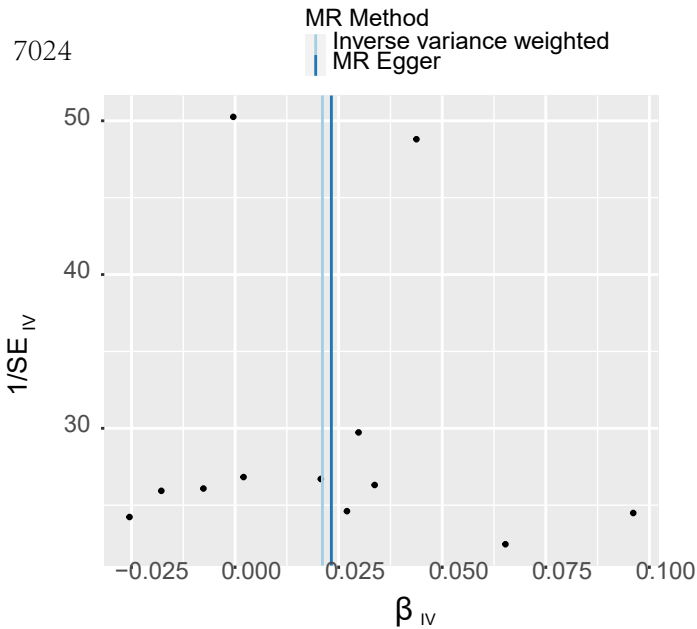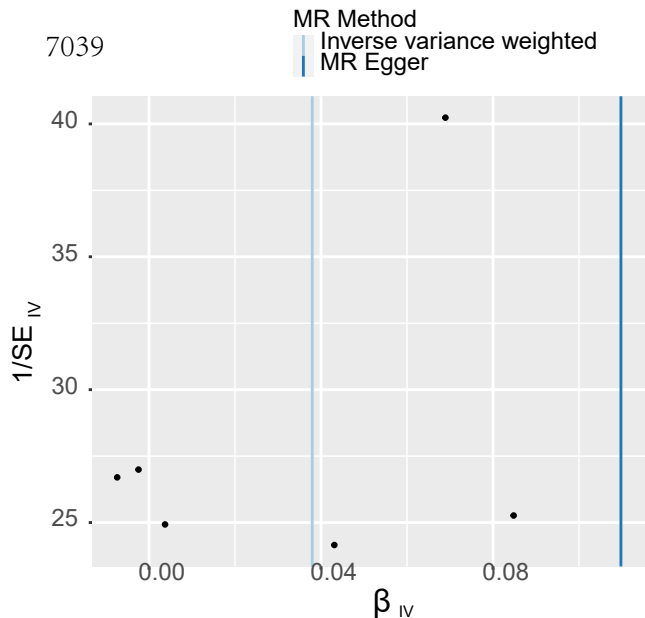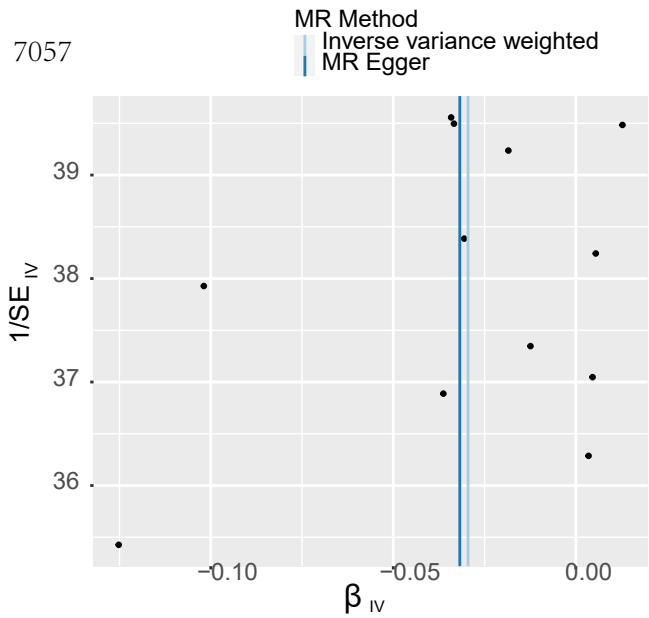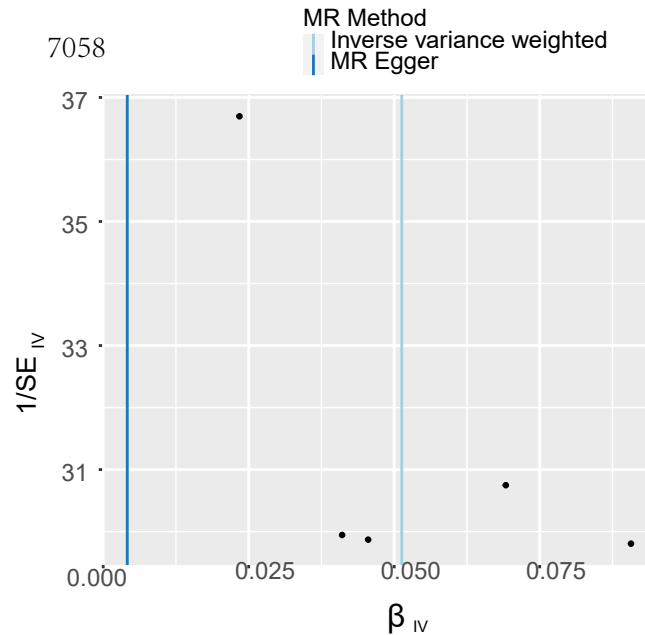

Supplement: Supplementary file 1 [file nutrients-15-04445-s001.zip › Supplementary materials 3/05.Funel plot for gut microbiota on APOA1.pdf]

# Gut microbiota genera and APOB

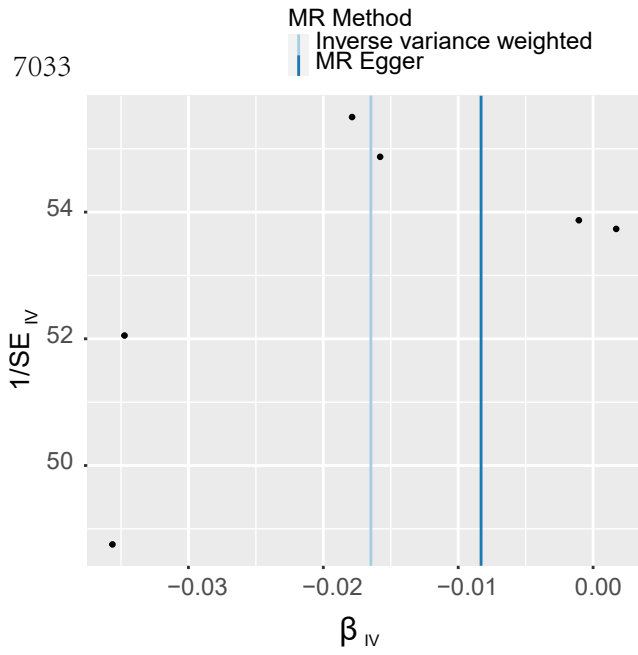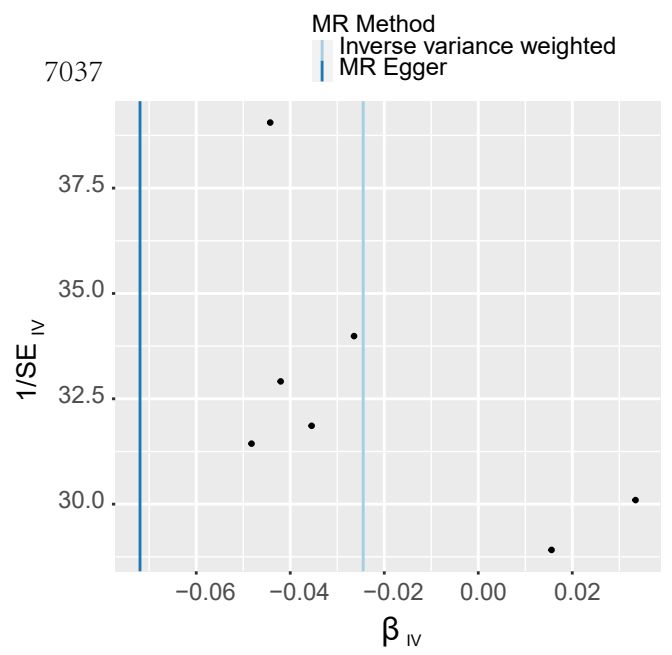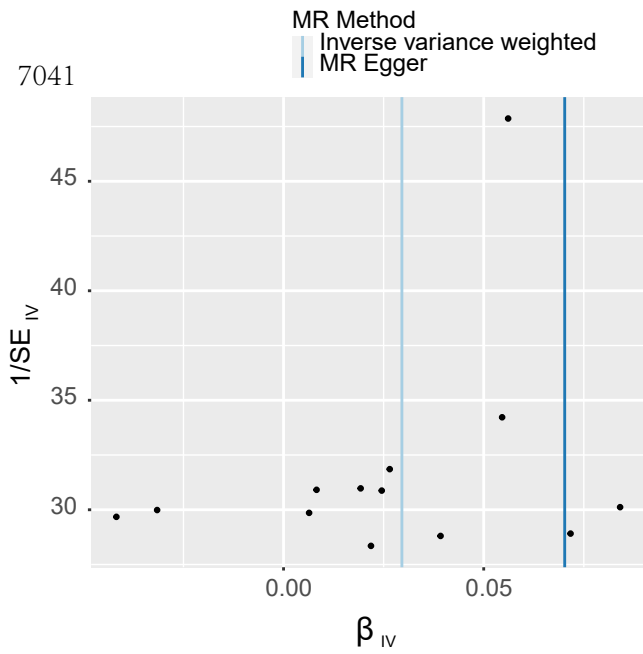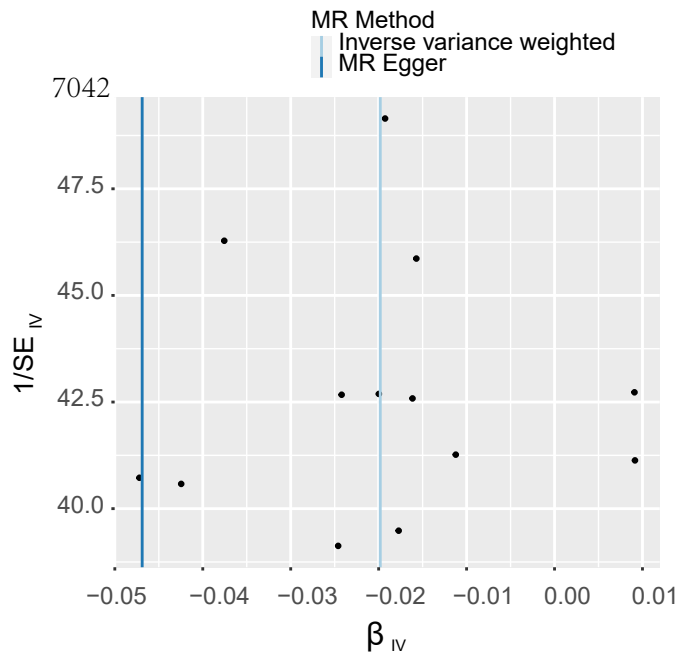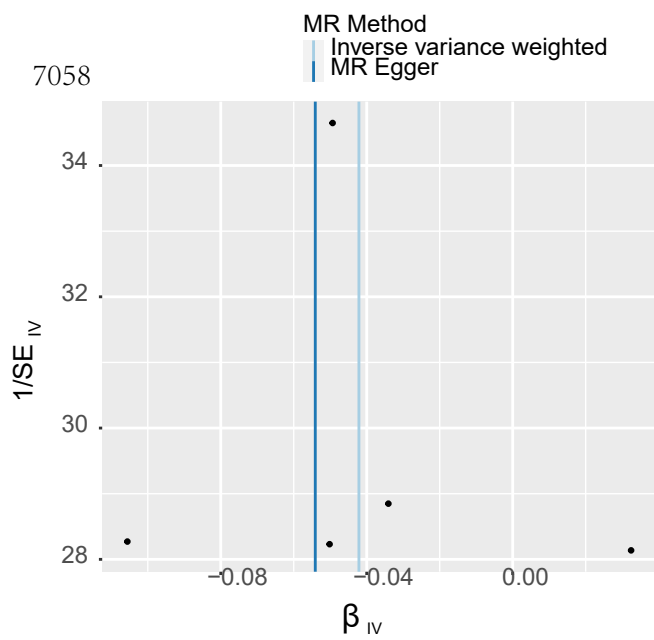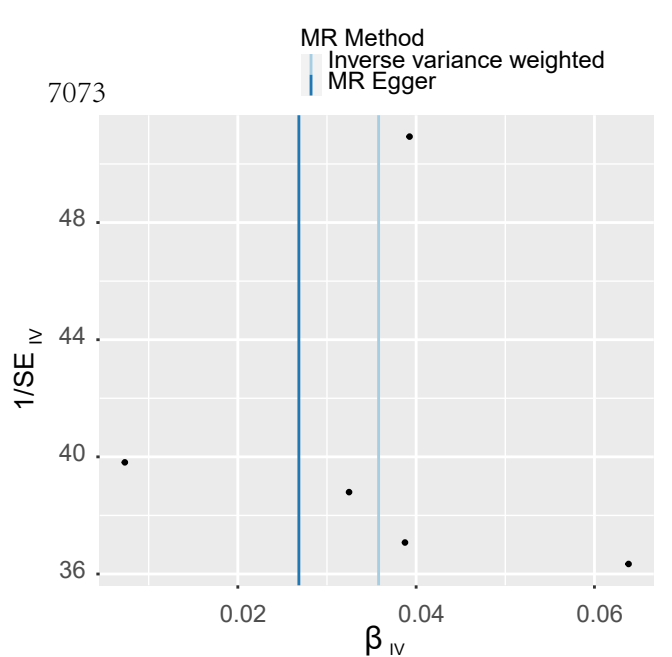

Supplement: Supplementary file 1 [file nutrients-15-04445-s001.zip › Supplementary materials 3/06.Funel plot for gut microbiota on APOB.pdf]
